# Supplementary material for: Hepatocytes Delete Regulatory T Cells by Enclysis, a CD4+ T Cell Engulfment Process
Source: Cell Rep. 2019 Nov 5;29(6):1610–1620.e4. doi: 10.1016/j.celrep.2019.09.068 (PMC7057271; doi:10.1016/j.celrep.2019.09.068)
Supplement: Document S1. Figures S1–S7 and Table S1 [file mmc1.pdf]

Cell Reports, Volume *29*

Supplemental Information

Hepatocytes Delete Regulatory T Cells by Enclysis, a CD4$^+$ T Cell Engulfment Process

Scott P. Davies, Gary M. Reynolds, Alex L. Wilkinson, Xiaoyan Li, Rebecca Rose, Maanav Leekha, Yuxin S. Liu, Ratnam Gandhi, Emma Buckroyd, Joe Grove, Nicholas M. Barnes, Robin C. May, Stefan G. Hubscher, David H. Adams, Yuehua Huang, Omar Qureshi, and Zania Stamataki

Suppl. Figure 1.

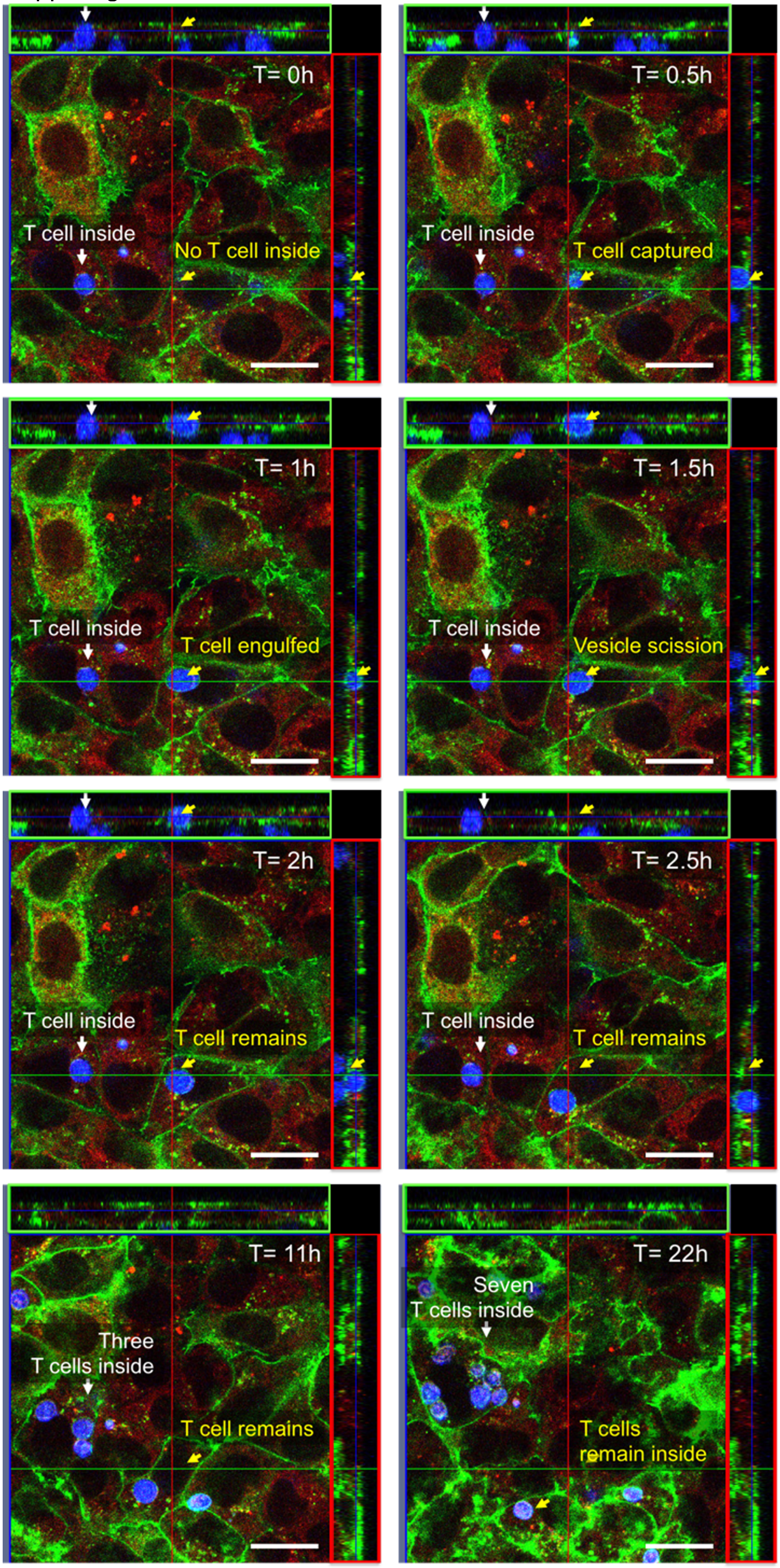

Suppl. Figure 1. Live CD4$^+$ T cells remained inside Huh-7 cells in vesicles that detached from the host cell membrane. Related to Figure 1. Time-lapse confocal imaging of Huh-7 cells (CMTPX, red cytoplasm) co-cultured with live CD4$^+$ T cells (BMQC, blue) over 22 hours. Huh-7 cell membranes were visualised with CD81-GFP (green). White arrow indicates a T cell that was fully internalised at the start of the imaging period (T=0). The consistent Z plane (blue line) was selected to confirm internalisation of the T cell, throughout the duration of the experiment. Orthogonal image coordinates (cross between green and red line) were set at the position where a new T cell appears, is captured and becomes engulfed within a vesicle that separated from the host cell membrane. Scale bars indicate 20 μm.

Suppl. Figure 2.

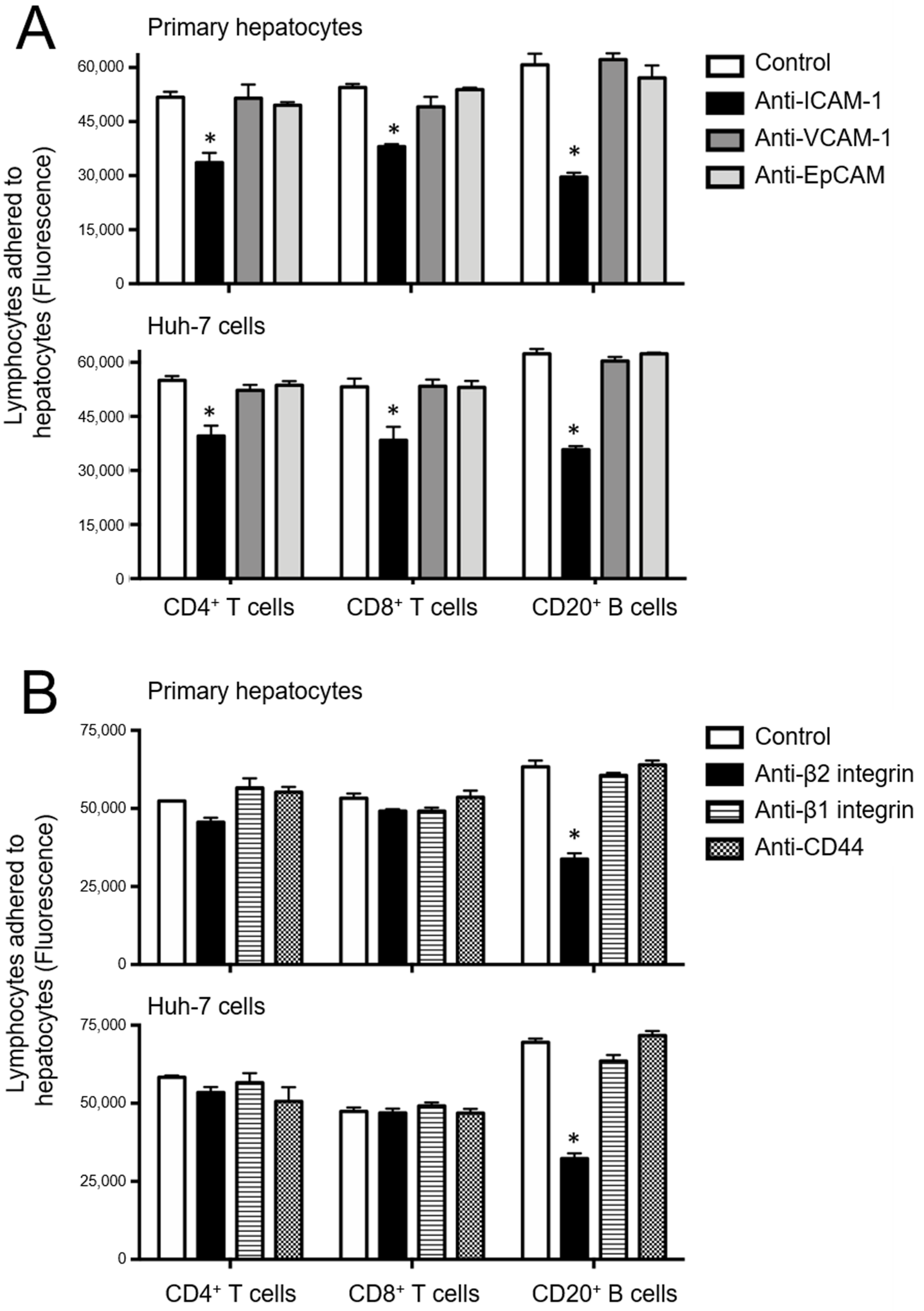

Suppl. Figure 2. Anti-ICAM-1 inhibits early lymphocyte adhesion to hepatocytes and Huh-7 cells. Related to Figure 2. Primary human hepatocytes or Huh-7 cells were seeded to confluence in 96-well plates and co-cultured with lymphocytes (CMFDA, green) for five minutes. **A.** Monolayers were treated with anti-ICAM-1, anti-VCAM-1 or anti-EpCAM or

isotype-matched control antibodies for 20 minutes before adding lymphocytes. **B.** Lymphocytes were treated with anti-β2 integrin, anti-β1 integrin, anti-CD44 or isotype-matched control antibodies before incubation with hepatocyte monolayers. Adhered lymphocytes persisting after washes were enumerated by total CMFDA fluorescence in replicate wells using a fluorescence plate reader. Data shown from four independent experiments with two primary hepatocyte donors and four lymphocyte donors. $*p < 0.05$, Student's non-parametric paired t test (Wilcoxon).

Suppl. Figure 3.

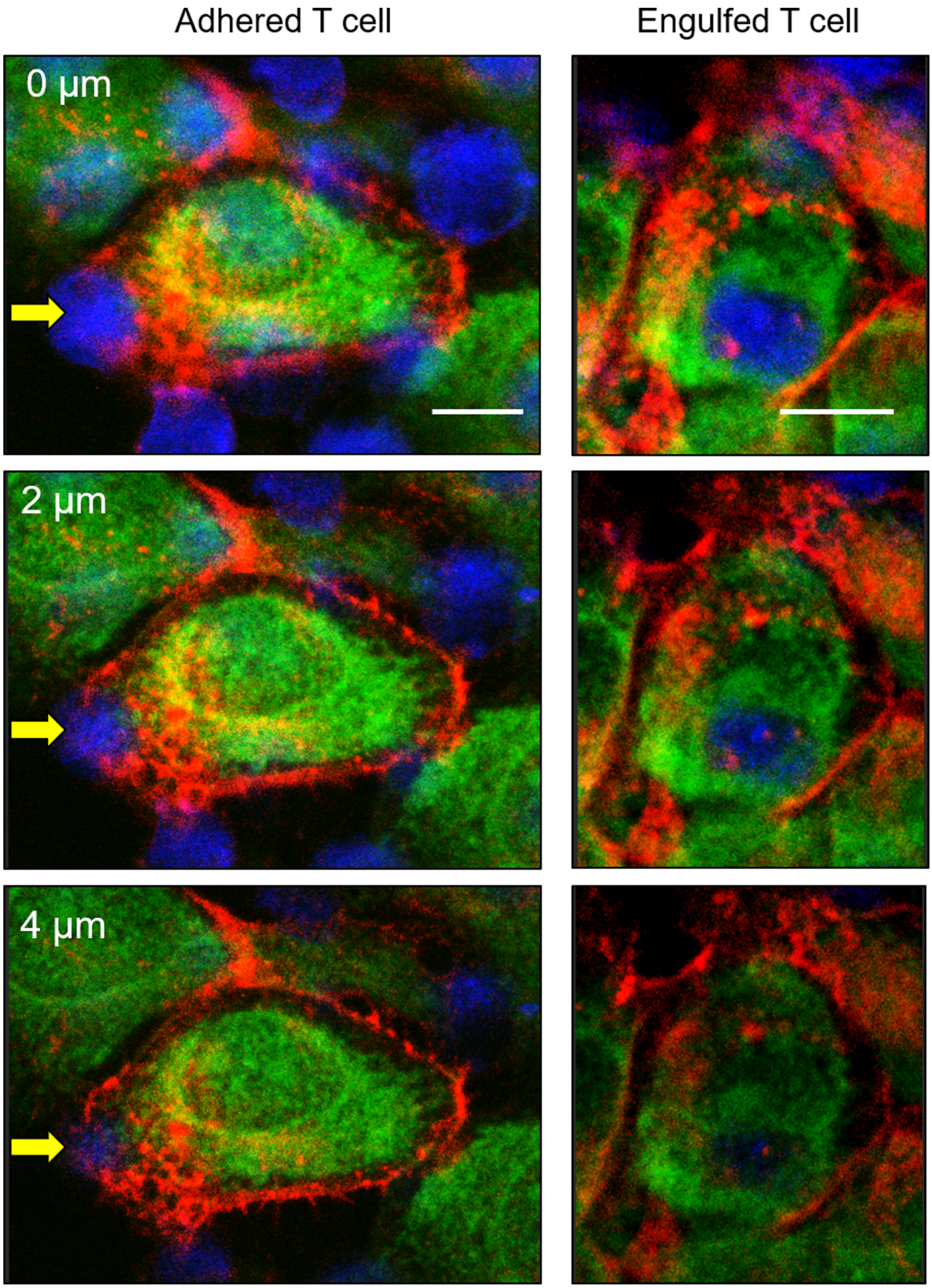

Suppl. Figure 3. Confocal z-stack images for ICAM-1 accumulation at the site of CD4^{+} T cell adhesion and engulfment. Related to Figure 2. Huh-7 cells (CMFDA, green) were

co-cultured with Jurkat T cells (BMQC, blue) for three hours, fixed and stained for ICAM-1 (anti-mouse-Alexa 594, red), then imaged by confocal microscopy using a x63 objective. Images shown are 2 μm apart. The yellow arrow indicates membrane ICAM-1 accumulations around a T cell that has not been completely internalised (left panel, adhered T cell). The right panel shows minimal ICAM-1 expression around the engulfed T cell. Scale bar represents 10 μm.

Suppl. Figure 4.

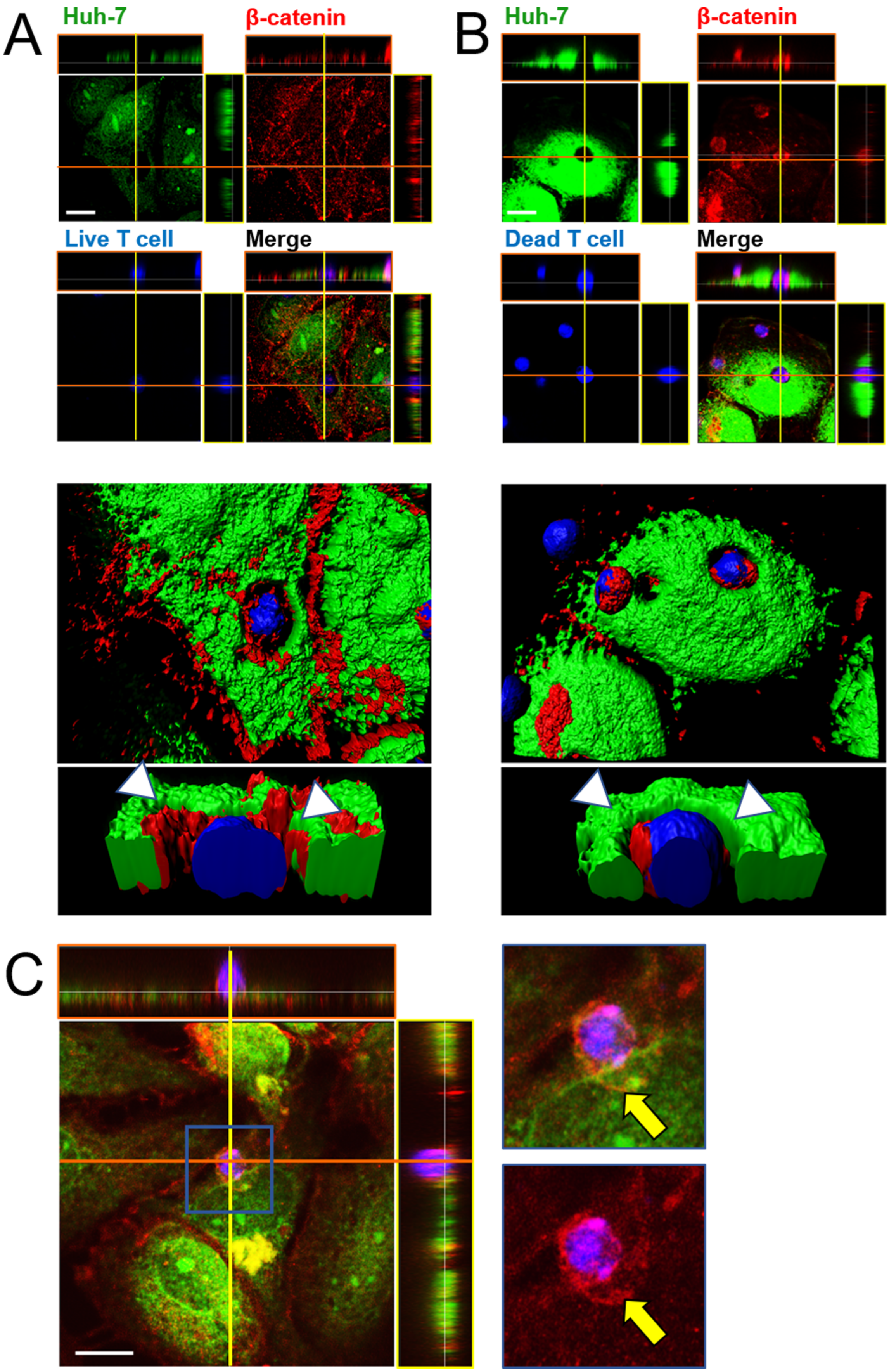

Suppl. Figure 4. Expression of β-catenin at the live T cell-containing vesicle and at the site of enclysis intermediates, but not in efferocytosis. **Related to Figure 4.** Huh-7 cells (CMFDA, green) were co-cultured with live or heat-killed Jurkat T cells (BMQC, blue) for three hours, fixed and stained for β-catenin (anti-mouse-Alexa 594, red), then imaged by confocal microscopy using a x63 objective. Non-rendered (Zeiss ZEN) and rendered (IMARIS) images are shown with white arrows showing the T cell-containing vesicle in **A.** enclysis and in **B.** efferocytosis. **C.** The presence β-catenin was also investigated in enclysis intermediates, where β-catenin accumulations were imaged on and around the adhered T cell (insets, yellow arrows). Scale bar represents 10 μm.

Suppl. Figure 5.

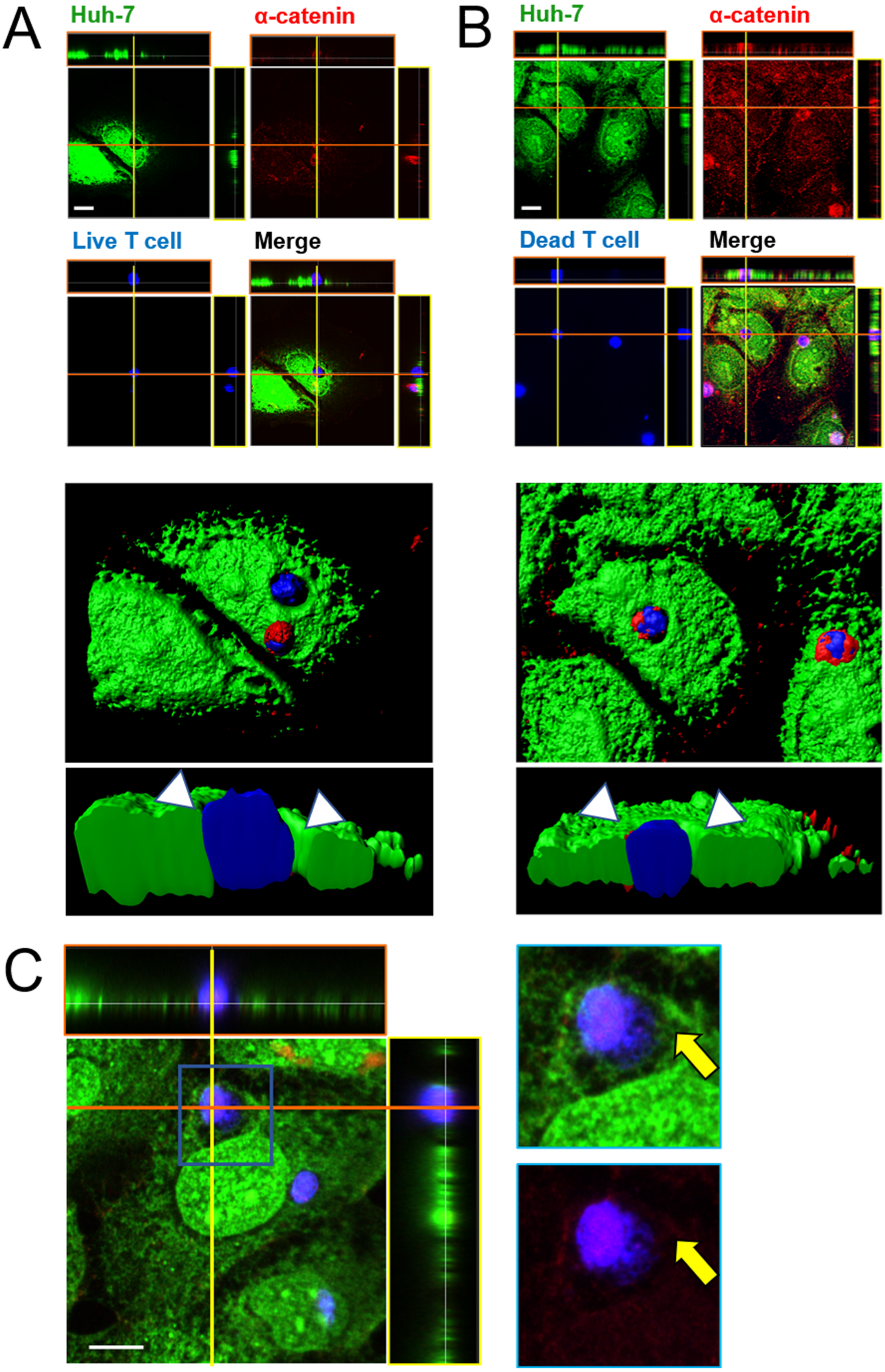

Suppl. Figure 5. α-catenin was not expressed at the site of enclysis intermediates and at the T cell-containing vesicle in enclysis or efferocytosis. **Related to Figure 4.** Huh-7 cells (CMFDA, green) were co-cultured with live or heat-killed Jurkat T cells (BMQC, blue) for three hours, fixed and stained for α-catenin (anti-mouse-Alexa 594, red), then imaged by confocal microscopy using a x63 objective. Non-rendered (Zeiss ZEN) and rendered (IMARIS) images are shown with white arrows showing the T cell-containing vesicle in **A.** enclysis and in **B.** efferocytosis. **C.** The presence α-catenin was also investigated in enclysis intermediates, where α-catenin did not accumulate around the adhered T cell (insets, yellow arrows). Scale bar represents 10 μm.

Suppl. Figure 6.

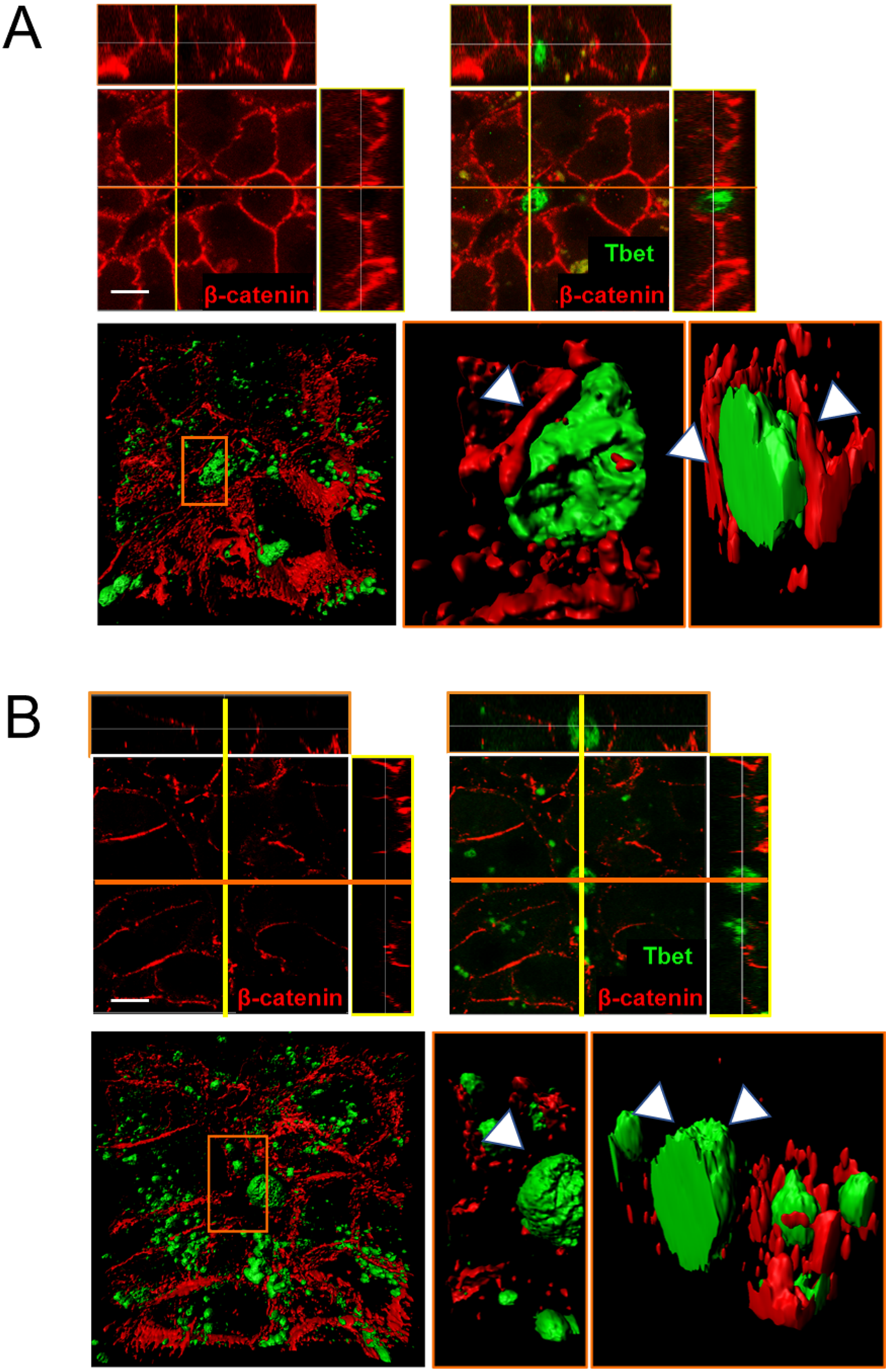

Suppl. Figure 6. Expression of β-catenin *in vivo* in end-stage liver disease. Related to Figure 4. Orthogonal confocal image of a 30 μm-thick section from formalin-fixed, paraffin-embedded tissue from a cirrhotic liver explant stained for β-catenin and $CD4^+$ T cell transcription factor Tbet. **A.** Hepatocyte containing a T cell where β-catenin molecules were observed at the enclytic vesicle (white arrows on 3-D rendered images by IMARIS). The hepatocyte membrane completely surrounds the enclosed T cell in the orthogonal view. **B.** Sinusoidal T cell for comparison, where there is no surrounding β-catenin association (white arrows on 3-D rendered images by IMARIS). Scale bar represents 10 μm.

Suppl. Figure 7.

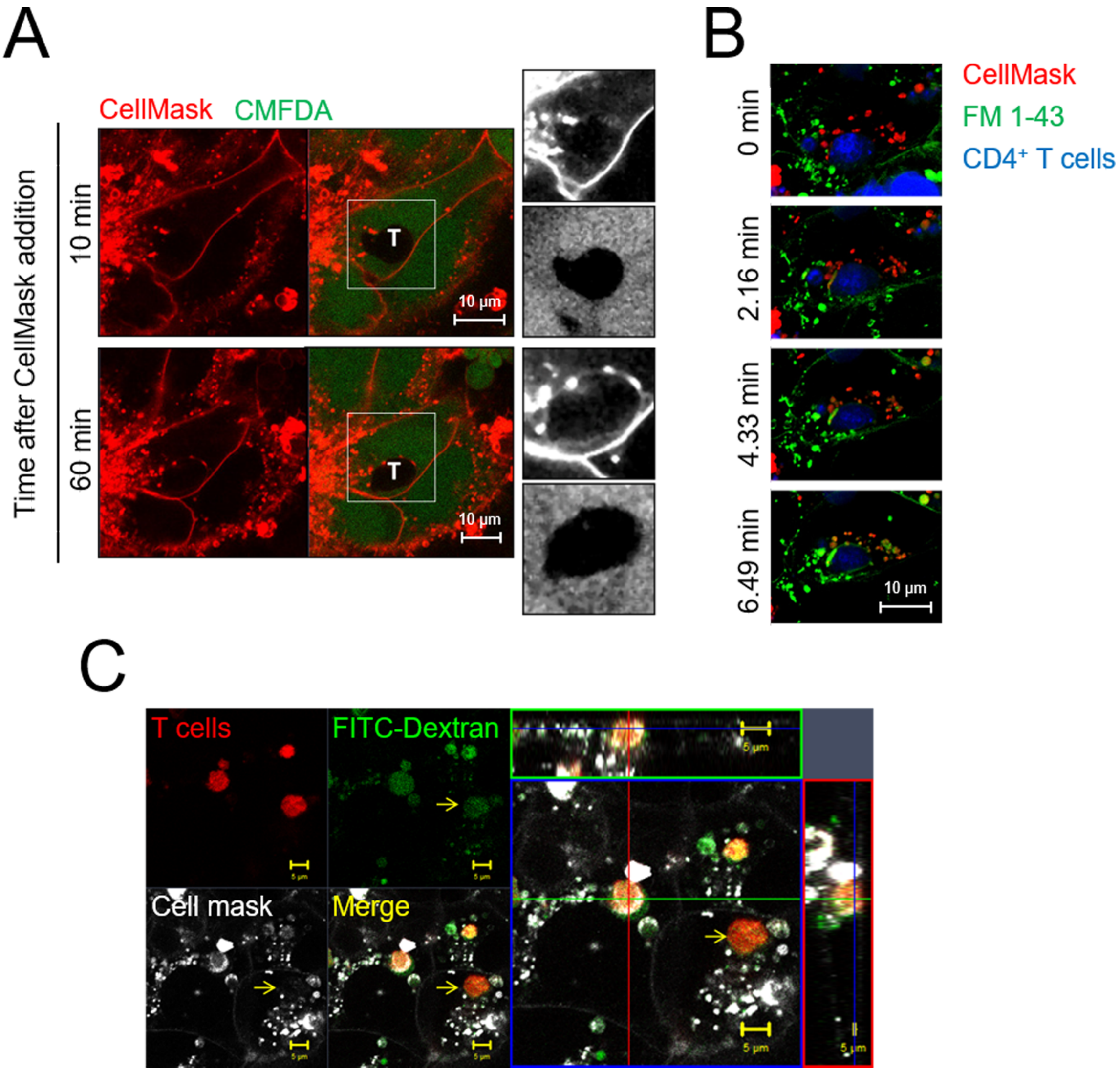

Suppl. Figure 7. Enclytic vesicles were connected to the endocytic pathway. Related to Figure 5. Live time-lapse confocal imaging detecting endocytic trafficking to the enclytic vesicle. **A.** Huh-7 cells (CMFDA, green) were co-cultured with $CD4^+$ T cells and enclytic vesicle detected (marked with a T), then CellMask™ Plasma Membrane Orange was applied to the culture media to reveal all accessible membranes. Imaged at 37°C, endosomes budding from the Huh-7 membrane (red) trafficked to the enclytic vesicle at 10 minutes following CellMask™ Plasma Membrane addition. Over time, endosomes fused with the enclytic vesicle membrane (60 minutes). **B.** Internalised T cell (BMQC, blue) was detected by CellMask™ Plasma Membrane Orange negative membrane (red) and FM 1-43 membrane dye (green). Within minutes (indicated), endocytic vesicles labelled by FM 1-43 trafficked to the T cell-containing vesicle and at six minutes the enclytic vesicle was labelled green. **C.** T cells inside Huh-7 cells were confirmed by lack of CellMask™ Plasma Membrane Deep Red staining. Fluorescent-labelled dextran (70kDa, FITC green) was added to the culture medium and the cells were imaged again following overnight incubation, when dextran had trafficked to the enclytic vesicle.

Suppl. Table 1. Patient information. Related to Figure 7. Age, sex and clinical information is given for patients used for immunohistochemistry measurements of enclysis. Patients were anonymised according to regulations approved by South Birmingham Research Ethics Committee. Donor livers: rejected for transplantation (non-cirrhotic). Autoimmune family disorders: autoimmune hepatitis (AIH), primary biliary cholangitis (PBC), primary sclerosing cholangitis (PSC). Metabolic injuries: alcohol liver disease (ALD), fatty liver disease (non-alcoholic steatohepatitis, NASH). Chronic viral infections: hepatitis B virus (HBV), hepatitis C virus (HCV). N/A: Not available.

	Donor	*AIH*	*PBC*	*PSC*	*ALD*	*NASH*	*HBV*	*HCV*
Cohort (n)	10	10	10	10	10	10	10	10
% female	N/A	50	0	30	40	50	10	10
Age Median	N/A	38.5	58	52	57	56	41.5	55.5
± SD		14.6	15.3	20.2	7.1	7.0	10.9	5.6
