# Supplementary material for: Hepatocytes Delete Regulatory T Cells by Enclysis, a CD4+ T Cell Engulfment Process
Source: Cell Rep. 2019 Nov 5;29(6):1610–1620.e4. doi: 10.1016/j.celrep.2019.09.068 (PMC7057271; doi:10.1016/j.celrep.2019.09.068)
Supplement: Document S2. Article plus Supplemental Information [file mmc9.pdf]

Cell Reports

Article

Hepatocytes Delete Regulatory T Cells by Enclysis, a CD4^{+} T Cell Engulfment Process

Graphical Abstract

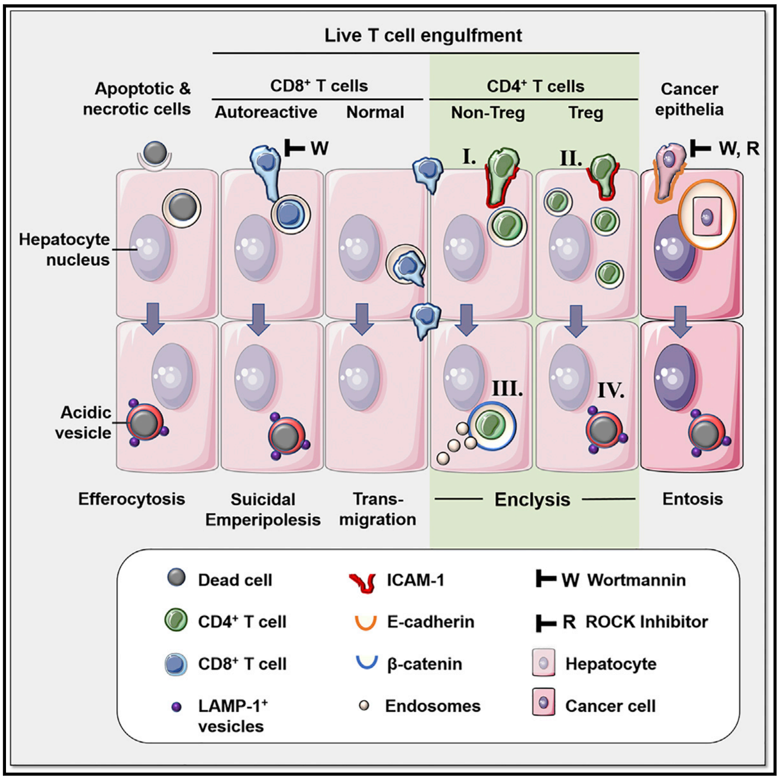

Authors

Scott P. Davies, Gary M. Reynolds, Alex L. Wilkinson, ..., Yuehua Huang, Omar Qureshi, Zania Stamataki

Correspondence

z.stamataki@bham.ac.uk

In Brief

Enclysis describes the enclosure of live CD4^{+} T cells in intracellular vesicles. Davies et al. show that hepatocytes engulf regulatory T (Treg) cells and preferentially delete them. Enclysis is distinct from entosis, efferocytosis, and suicidal emperipolesis and may be targeted to control T cell populations in the liver during inflammation.

Highlights

- Hepatocytes engulf live CD4^{+} T cells, with a preference for regulatory cells
- We called this process enclysis and compared it with known cell-in-cell processes
- ICAM-1 and β-catenin accumulated at the point of T cell engulfment
- Enclysis was seen in health, in liver cancer, and especially in autoimmune disorders

Davies et al., 2019, Cell Reports *29*, 1610–1620
November 5, 2019 © 2019 The Author(s).
https://doi.org/10.1016/j.celrep.2019.09.068

OPEN
ACCESS
CellPress

Cell Reports
Article

Hepatocytes Delete Regulatory T Cells by Enclysis, a CD4^{+} T Cell Engulfment Process

Scott P. Davies,[1] Gary M. Reynolds,[1,2] Alex L. Wilkinson,[1] Xiaoyan Li,[1,3] Rebecca Rose,[1] Maanav Leekha,[1] Yuxin S. Liu,[4] Ratnam Gandhi,[1] Emma Buckroyd,[1] Joe Grove,[5] Nicholas M. Barnes,[6] Robin C. May,[7] Stefan G. Hubscher,[1,2,8] David H. Adams,[1,2] Yuehua Huang,[3] Omar Qureshi,[9,10] and Zania Stamataki[1,2,11,12,*]

[1]Institute of Immunology and Immunotherapy, Centre for Liver and Gastrointestinal Research, University of Birmingham, Birmingham, UK
[2]NIHR Birmingham Liver Biomedical Research Centre, University Hospitals Birmingham NHS Foundation Trust, Birmingham, UK
[3]Department of Infectious Diseases and Guangdong Provincial Key Laboratory of Liver Disease Research, The Third Affiliated Hospital of Sun Yat-sen University, Guangzhou, China
[4]Institute of Inflammation and Aging, University of Birmingham, Birmingham, UK
[5]Institute of Immunity and Transplantation, Division of Infection and Immunity, University College London, London, UK
[6]Neuropharmacology Research Group, Institute of Clinical Sciences, University of Birmingham, Birmingham, UK
[7]Institute of Microbiology and Infection and School of Biosciences, University of Birmingham, Birmingham, UK
[8]Department of Cellular Pathology, University Hospitals Birmingham NHS Foundation Trust, Birmingham, UK
[9]Celentyx Ltd., Birmingham Research Park, Birmingham B15 2SQ, UK
[10]Celentyx Ltd., BioEscalator Innovation Building, Oxford OX3 7FZ, UK
[11]Twitter: @ZaniaStamataki
[12]Lead Contact
*Correspondence: z.stamataki@bham.ac.uk
https://doi.org/10.1016/j.celrep.2019.09.068

SUMMARY

CD4^{+} T cells play critical roles in directing immunity, both as T helper and as regulatory T (Treg) cells. Here, we demonstrate that hepatocytes can modulate T cell populations through engulfment of live CD4^{+} lymphocytes. We term this phenomenon enclysis to reflect the specific enclosure of CD4^{+} T cells in hepatocytes. Enclysis is selective for CD4^{+} but not CD8^{+} cells, independent of antigen-specific activation, and occurs in human hepatocytes *in vitro*, *ex vivo*, and *in vivo*. Intercellular adhesion molecule 1 (ICAM-1) facilitates T cell early adhesion and internalization, whereas hepatocytes form membrane lamellipodia or blebs to mediate engulfment. T cell internalization is unaffected by wortmannin and Rho kinase inhibition. Hepatocytes engulf Treg cells more efficiently than non-Treg cells, but Treg cell-containing vesicles preferentially acidify overnight. Thus, enclysis is a biological process with potential effects on immunomodulation and opens a new field for research to fully understand CD4^{+} T cell dynamics in liver inflammation.

INTRODUCTION

The liver is an immune-tolerizing environment that, unlike other organs, enables successful transplantation without histocompatibility restrictions (with the help of immunosuppression) (Calne et al., 1969; Levitsky and Feng, 2018). Liver tolerance is perturbed in chronic inflammation caused by autoimmune disorders, viral infection, or metabolic injuries (Grant and Liberal, 2017; Heymann and Tacke, 2016). Understanding immune cell interactions with liver cells is critical to design effective therapies for chronic liver diseases, which can lead to fibrosis, cirrhosis, and end-stage liver diseases requiring a transplant.

Cell-in-cell structures formed by live-cell engulfment have been observed in multiple cell types (Overholtzer and Brugge, 2008). Bertolino et al. (1998) reported that recently activated autoreactive CD8^{+} T cells invade hepatocytes expressing their cognate antigen and are rapidly degraded by wortmannin-sensitive "suicidal emperipolesis" in autoimmune mice (Benseler et al., 2011). In contrast, non-autoreactive CD8^{+} T cells are not deleted in the liver (Benseler et al., 2011). The nature of CD4^{+} T helper cell-hepatocyte interactions has not been described previously beyond the effects of hepatocyte co-culture on T cell immune function (Bertolino et al., 1998; Burghardt et al., 2013, 2014; Qian et al., 2001; Sana et al., 2014; Wiegard et al., 2007). Here we describe a mechanistically distinct process in which CD4^{+} T cells, but not CD8^{+} T cells or B cells from the same donors, internalized into hepatocytes and hepatocyte cancer-derived cell lines *in vitro*, *ex vivo*, and *in vivo*.

Hepatocytes are epithelia that comprise over 80% of the liver mass and are organized in roughly two-cell-thick cords, flanked by specialized fenestrated endothelium that lines the liver sinusoids (Shetty et al., 2018). Immune cells passing through the sinusoidal channels under shear flow conditions do not need to migrate through endothelia to interact with the underlying hepatocytes (Warren et al., 2006). Instead, in elegant intravital microscopy studies by Guidotti et al. (2015), it has been shown that reaching through endothelial fenestrations is sufficient for cytotoxic CD8^{+} T cells to detect and delete hepatitis B antigen-expressing hepatocytes. Trans-endothelial migration is a highly regulated process that allows T cells to enter the parenchyma during inflammation, requiring prior activation of the sinusoidal endothelium. We have previously demonstrated that regulatory T (Treg) cells transmigrate via the trans-cellular route (Shetty et al., 2011) and require endothelial activation by both

 © 2019 The Author(s).
This is an open access article under the CC BY license (http://creativecommons.org/licenses/by/4.0/).

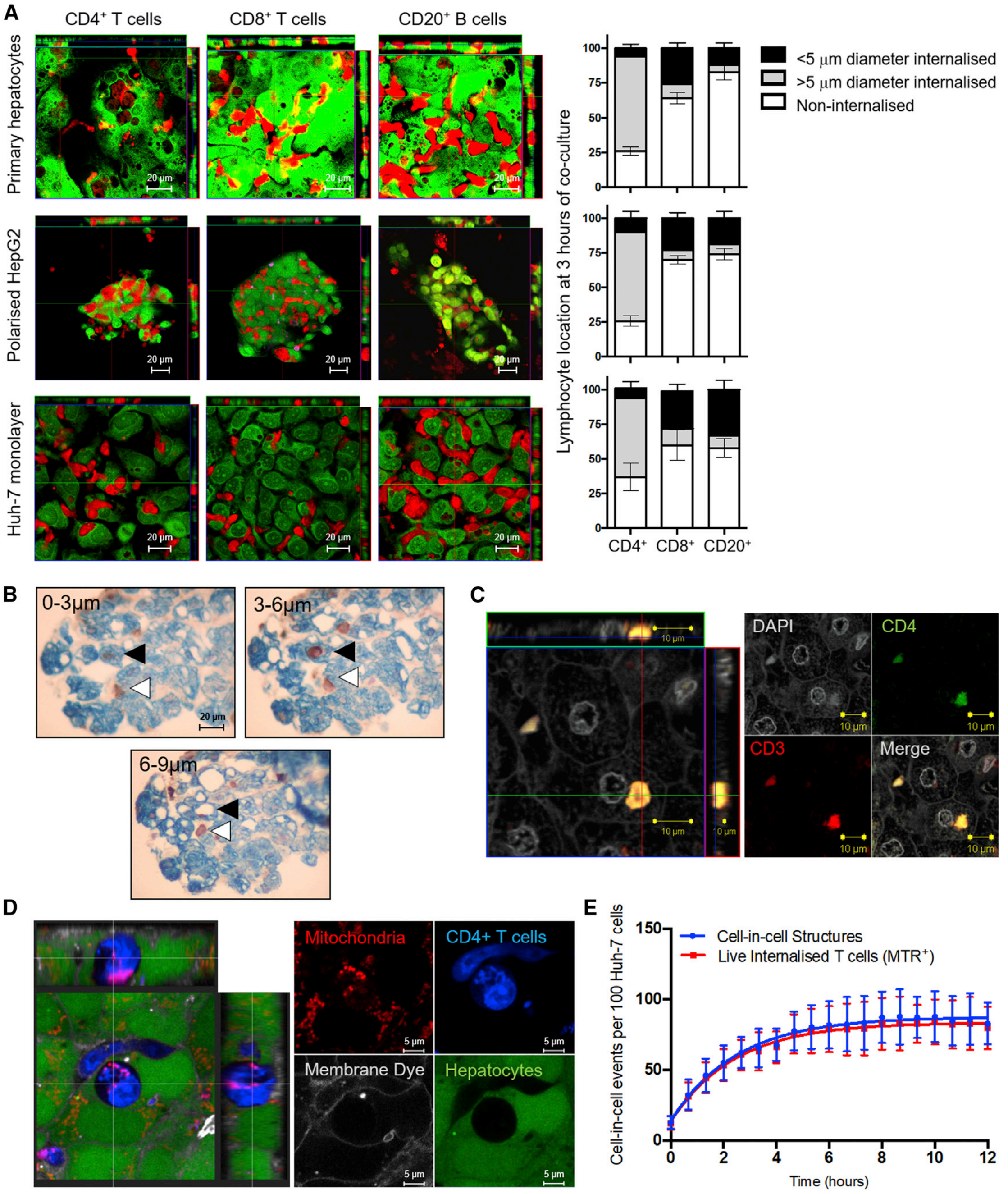

Figure 1. Live CD4$^+$ T Cells Internalized into Hepatocytes and Hepatocyte Cancer Cell Lines

Figure360 For a Figure360 author presentation of this figure, see https://doi.org/10.1016/j.celrep.2019.09.068.

(A) CD4$^+$ T cells, CD8$^+$ T cells, and CD20$^+$ B cells (CMTPX, red) were co-cultured with hepatocytes, HepG2 cell spheroids polarized to 80% (measured by MRP-2 staining), or a monolayer of Huh-7 cells (5-chloromethylfluorescein diacetate [CMFDA], green) for 3 h. CD4$^+$ T cells were found predominantly in hepatocytes in all cases (gray bars), whereas internalization events in CD8$^+$ T cell and B cell co-cultures involved mainly cell debris smaller than 5 µm in diameter (black bars). Non-internalized lymphocytes are shown as white bars. Error bars demonstrate SD from four independent experiments.

(B) Biotinylated peripheral blood-derived CD4$^+$ T cells were added to human liver biopsies and co-cultured for 3 h. The T cells (streptavidin/horseradish peroxidase [HRP], and 3,3′-diaminobenzidine [DAB], brown) transmigrated and were found in sinusoids (white arrowheads) or internalized into hepatocytes (pan-cytokeratin, blue), shown by black arrowheads, in 3-µm-thick serial sections.

(C) Confocal z stack showing a CD4$^+$ CD3$^+$ T cell in a hepatocyte in a patient liver with end-stage disease. Anti-rabbit CD3-Alexa 594, red; anti-mouse CD4-Alexa 488, green; DAPI, white.

(legend continued on next page)

OPEN
ACCESS
CellPress

tumor necrosis factor alpha (TNF-α) and interferon γ (IFN-γ) (Oo et al., 2010).

In this study, we investigated $CD4^+$ T cells in the tissue parenchyma and established their fate, which differed for Treg cells and non-Treg cells. We focused on the cell biology of T cell-hepatocyte interactions and demonstrate that T cell engulfment by hepatocytes requires action from both cell types. This process was distinct to (1) phagocytosis of apoptotic and necrotic cells (efferocytosis) (Davies et al., 2018), (2) deletion of autoreactive $CD8^+$ T cells by suicidal emperipolesis (Benseler et al., 2011), and (3) the process of epithelial homotypic cell invasion prominent in metastatic cancers, known as entosis (Overholtzer et al., 2007).

We termed the engulfment of live $CD4^+$ T cells by hepatocytes enclysis (Εγκλείω ἐγ- (ἐν-) + κλείω, to enclose, to confine, to keep in captivity). Like entosis, enclysis may lead to deletion of the internalized T cell, but this was not always the case. Our experiments indicate that the outcome of enclysis is influenced by the identity of the cell cargo because Treg cells were deleted more frequently than non-Treg cells. Unlike previously described cell-in-cell mechanisms, enclysis involves membrane bleb formation and shares features with macropinocytosis (Mercer and Helenius, 2009; Swanson, 2008; Swanson and Watts, 1995). This phenomenon provides a further aspect of immune regulation in the liver, which could be exploited in drug discovery.

RESULTS

Hepatocytes Preferentially Engulf Live $CD4^+$ T Cells but Not $CD8^+$ T Cells or B Lymphocytes

To understand the mode of lymphocyte interaction with hepatocytes, primary human hepatocytes (PHHs) from liver explants and peripheral blood-derived lymphocyte subsets were labeled with CellTracker dyes and co-cultured for 3 h. $CD8^+$ T cells and B cells spontaneously migrated between hepatocytes (paracellular migration), whereas $CD4^+$ T cells were documented inside hepatocytes within large vesicles (Figure 1A). This was also the case when lymphocytes were co-cultured with polarized HepG2-CD81 hepatoblastoma 3D spheroids, which form functional $MRP2^+$ bile canaliculi (Mee et al., 2009), and with Huh-7 hepatocellular carcinoma cells, which form a monolayer in 2D culture. Hepatocytes and hepatic cancer cell lines are competent efferocytes, readily engulfing apoptotic cell debris *in vitro* (Davies et al., 2018). To take this into account, we measured the diameter of engulfed cellular material by hepatocytes in our co-cultures; most intracellular material in the $CD8^+$ T cell and B cell co-cultures was less than 5 μm in diameter, consistent with digested debris. Conversely, most internalized material in the $CD4^+$ T cell co-culture was around 10 μm in diameter, the size of intact lymphocytes.

We previously showed that T cells migrate through sinusoidal endothelia using trans-cellular pores (Shetty et al., 2011). Time-lapse confocal imaging confirmed that T cells in hepatocytes remained internalized for over 22 h; therefore, T cell engulfment by hepatocytes did not lead to trans-cellular migration (Figure S1; Videos S1, S2, S3, S4, S5, and S6).

To investigate whether hepatocytes would engulf T cells *ex vivo*, stimulated $CD4^+$ T cells were biotinylated and co-cultured for 3 h with biopsies from patients with end-stage liver disease (Figure 1B). Immunohistochemical staining of serial sections of formalin-fixed, paraffin-embedded tissue revealed spontaneous trans-endothelial migration and engulfment of the $CD4^+$ T cells by $cytokeratin^+$ hepatocytes. We also detected the presence of $CD4^+CD3^+$ T cells in hepatocytes *in vivo* in explants from patients with end-stage liver diseases (Figure 1C).

We next set out to study whether $CD4^+$ T cells in hepatocytes were dying, destined for deletion akin to efferocytosis. Viable cells have mitochondria with active membrane potential that can be visualized by the cell-permeable dye MitoTracker Red. Complete internalization of CellTracker-labeled $CD4^+$ T cells by Huh-7 cells was confirmed by lack of staining with CellMask Plasma Membrane, a non-permeable dye added to the cell culture medium. Our imaging experiments showed that T cells completely engulfed by hepatocytes were viable 24 h following internalization (Figure 1D). Time-lapse imaging confirmed that internalized T cells continued to have active mitochondria 12 h following internalization (Figure 1E). Taken together, our studies show that hepatocytes and hepatocyte cancer cell lines preferentially engulf $CD4^+$ T cells, which remained viable in hepatocytes.

ICAM-1 Is Involved in $CD4^+$ T Cell Engulfment by Hepatocytes and Huh-7 Cells

Interaction of lymphocytes with hepatocytes *in vitro* can be divided into three phases: early adhesion (5 min), firm binding and/or para-cellular positioning (30 min), and spontaneous transmigration (60 min) (Figure 2A). To investigate adhesion molecules involved in lymphocyte-hepatocyte interactions, we incubated PHHs, Huh-7 cells, or peripheral blood-derived lymphocyte subsets with blocking antibodies before co-culture and measured lymphocyte adhesion (Figure S2). Anti-ICAM-2, anti-vascular cell adhesion molecule 1 (VCAM-1), and anti-epithelial cell adhesion molecule (EpCAM) antibodies failed to inhibit lymphocyte adhesion to PHH and Huh-7 cells *in vitro*; however, anti-ICAM-1 antibodies inhibited $CD4^+$ T cell binding in a time-dependent manner (Figure 2B; Figure S2A). We were unable to perturb T cell-hepatocyte adhesion by inhibition of the ICAM-1 binding partner β2-integrin on T cells; however, this was possible for B lymphocytes (Figure S2B). ICAM-1 was therefore involved in the early stage of T cell adhesion to

(D) Confocal image of a $CD4^+$ T cell (BMQC, blue) internalized into a Huh-7 cell (CMFDA, green), showing active mitochondria in live cells 24 h following co-culture (MitoTracker Red). The internalized T cell was not accessible to the membrane dye (CellMask Plasma Membrane, white), which was present in the culture medium.

(E) Kinetics profile (blue) of $CD4^+$ T cell capture by Huh-7 cells as measured by time-lapse microscopy using a CQ1 high-content benchtop microscope. The proportion of internalized T cells that remained metabolically active (MitoTracker Red^+, red line) throughout the time course is indicated.

Data shown are mean ± SD of triplicate wells (three fields per well) and are representative of two independent experiments.

See also Figure S1 and Videos S1, S2, S3, S4, S5, and S6.

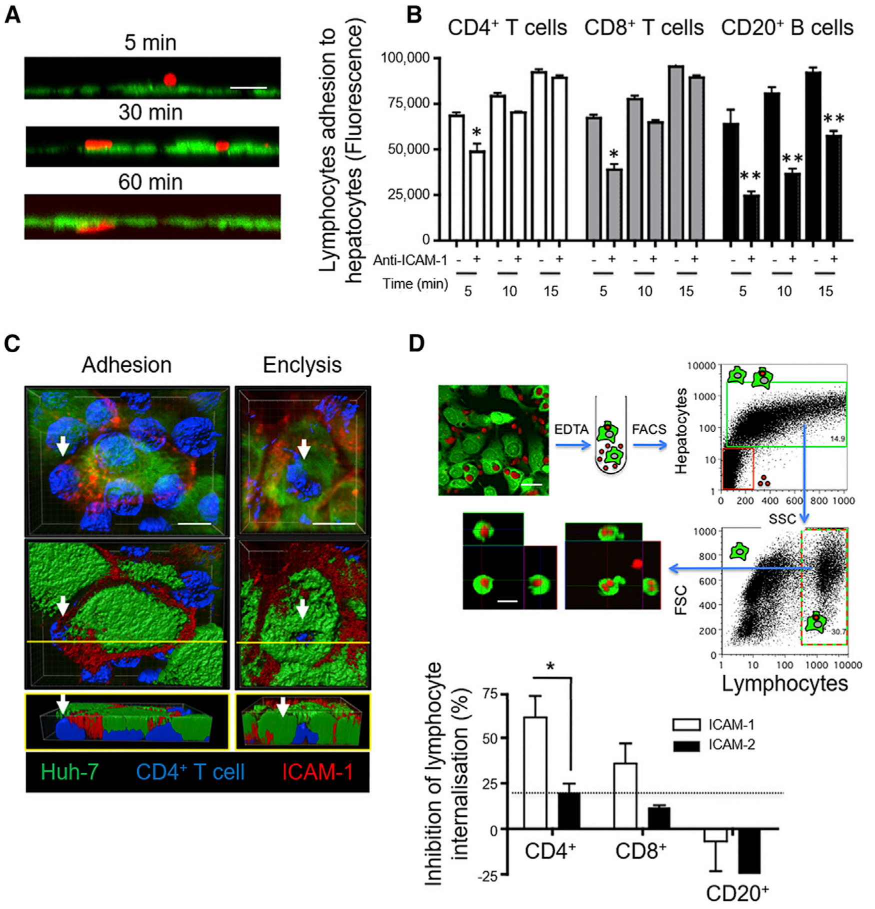

Figure 2. Anti-ICAM-1 Inhibits Early Adhesion and Enclysis of CD4+ T Cells

Huh-7 cells (CMFDA, green) were co-cultured with lymphocytes.

(A) Removing lymphocytes (CMPTX, red) by washes at the indicated times allowed measurement of early adhesion (5 min), firm binding (30 min), and trans-epithelial migration (60 min) by confocal microscopy.

(B) Anti-ICAM-1 or isotype-matched control antibodies were added to a confluent Huh-7 monolayer before lymphocyte co-culture, and adhered lymphocytes (CMFDA, green) were washed away at the indicated times. Adhered lymphocytes were enumerated by total CMFDA fluorescence in replicate wells of a 96-well plate.

(C) Huh-7 cells (CMFDA, green) were co-cultured with Jurkat T cells (BMQC, blue) for 3 h, fixed, and stained for ICAM-1 (anti-mouse Alexa 594, red) and then imaged by confocal microscopy. The scale bars represent 20 μm.

(D) Huh-7 cells (CMFDA, green) were co-cultured with lymphocytes (CMTPX, red) in the presence of anti-ICAM-1, anti-ICAM-2, or isotype-matched control antibodies for 3 h and dislodged from plastic using EDTA. Huh-7 cells containing a lymphocyte were sorted by flow cytometry, and internalization was confirmed by confocal microscopy. Inhibition of lymphocyte internalization was enumerated compared with an isotype-matched control. The dotted line indicates assay variation cutoff across all conditions (±22%).

Error bars indicate SD from at least three independent experiments. $^{*}p < 0.05$, $^{**}p < 0.01$, $^{***}p < 0.001$; Student's non-parametric paired t test (Wilcoxon). See also Figures S2 and S3.

hepatocytes but likely continued to be important beyond 15 min (firm binding) for B cells (Figure 2B). Confocal imaging confirmed that ICAM-1 aggregated around the adhered T cells at the site of engulfment but was not enriched around the enclytic vesicle (Figure 2C; Figure S3).

To investigate the role of ICAM-1 during CD4+ T cell engulfment, we devised a medium-throughput flow cytometry-based assay (Figure 2D). Huh-7 monolayers were pre-treated with neutralizing antibodies to ICAM-1, ICAM-2, or isotype-matched controls before co-culture with T cells for 3 h. Cells were dislodged from plastic using EDTA and filtered to form a single-cell suspension, and cell-in-cell structures were enumerated by flow cytometry. Huh-7 cells associating with large lymphocytes were determined by size, granularity, and CellTracker dye fluorescence intensity. Flow-sorted cells were imaged by confocal microscopy to confirm cell-in-cell structures. In these experiments, anti-ICAM-1, but not anti-ICAM-2, inhibited CD4+ T cell engulfment by hepatocytes. Of note, a small proportion of cell-in-cell structures in these assays reflects efferocytosis of apoptotic cells (as seen in Figure 1A), which explains the small percentage of CD8+ T cells and B cells associating with hepatocytes by flow cytometry. There was no effect on CD8+ T cell or B cell association with hepatocytes, which were not different from the assay cutoff, as determined by isotype-matched controls.

T Cell Internalization by Hepatocytes Was Distinct from Efferocytosis and Resembled Macropinocytosis

It is possible that hepatocytes mistake CD4+ T cells for apoptotic or necrotic cells and therefore attempt their clearance by efferocytosis. Efferocytosis is the phagocytosis of dead and dying cells that involves membrane ruffle formation, which aids cell capture and engulfment (Nakaya et al., 2006). We investigated the membrane alterations involved in capture of CD4+ T cells from peripheral blood by scanning electron microscopy (white arrows, Figure 3A). Huh-7 cells developed fine cellular ruffles following adhesion of apoptotic (note the smooth membrane) and necrotic (porous membrane) T cells, whereas firm adhesion of live CD4+ T cells resulted in abundant bleb formation surrounding the live T cell. In the time-lapse confocal experiments described in Figure S1, the capture of T cells by enclysis shows lamellipodium-like structures (Videos S1, S2, S3, and S4). Lamellipodia were more frequently observed in time-lapse microscopy, with membrane blebs detectable in 10%–30% of cases in various experiments. Therefore, the process of live T cell engulfment resembled macropinocytosis, which uses lamellipodia and blebs for engulfment of large solutes in extracellular liquid (Mercer and Helenius, 2009).

Hepatocytes and hepatocyte cancer cell lines are efficient efferocytes, with over 90% capable of efferocytosis (Davies et al., 2018); a fraction, however, were able to engulf live

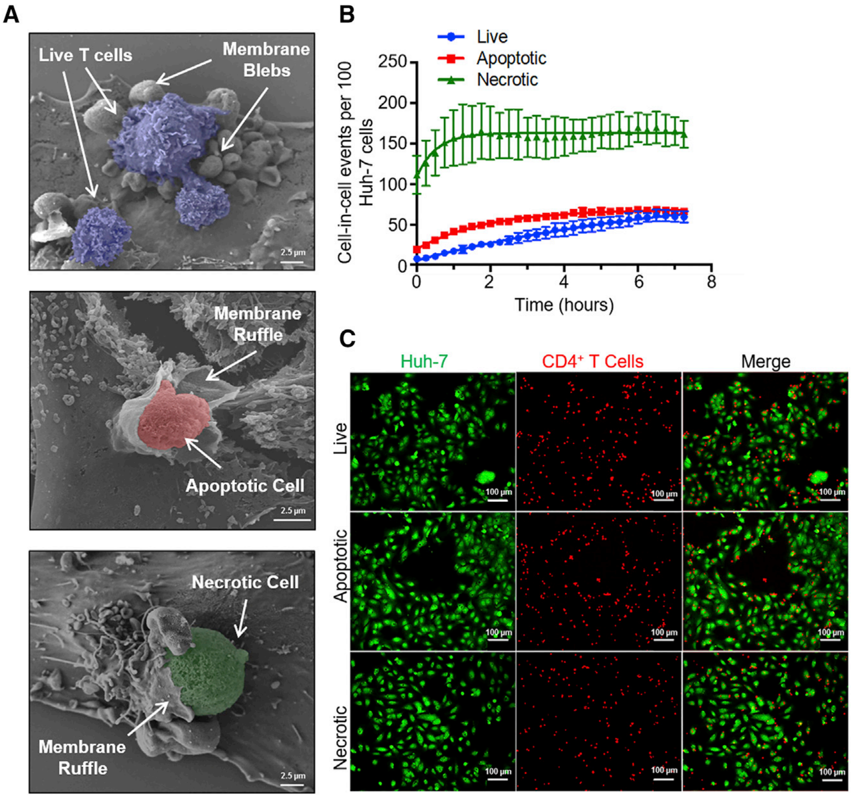

Figure 3. Enclysis Involves Membrane Blebbing and Is Distinct from Efferocytosis

(A) Live, apoptotic (staurosporine-treated), or necrotic (heat-killed) T cells were co-cultured with Huh-7 cells for 3 h, fixed, and imaged by scanning electron microscopy. Images show primary CD4$^+$ T cells isolated from peripheral blood. White arrows show membrane alterations on Huh-7 cells.

(B) Kinetics profiles of live (blue), apoptotic (red), or necrotic (green) T cell capture, determined by time-lapse imaging, following co-culture with Huh-7 cells over 7 h and imaged every 15 min using a CQ1 high-content benchtop microscope. Cell-in-cell structures were enumerated in three fields of view from triplicate wells over time and plotted, with error bars showing SD.

(C) Representative images showing capture of live, apoptotic, and necrotic CD4$^+$ T cells (BMQC, red) by Huh-7 cells (CMFDA, green) at time (t) = 7 h.

T cells in time-lapse experiments using fluorescence microscopy (Figures 3B and 3C). Necrotic T cell capture was the fastest process ($t_{1/2}$ = 31.8 min). Live T cell engulfment was slow ($t_{1/2}$ = 321 min), and a maximum of 40% of Huh-7 cells captured a live T cell. The kinetics of enclysis are therefore slower and less efficient than efferocytosis *in vitro*.

T Cell Internalization into Hepatocytes Was Distinct from Entosis and Suicidal Emperipolesis

Cell-in-cell structures involving live cargo have been brought to the fore in seminal work by Overholtzer et al. (2007), characterizing entosis. Entosis was originally described as the process of cancer epithelial cell internalization that occurs during matrix detachment or cell division, where cells pull at each other via adhesion molecules, resulting in neighboring cell invasion (Durgan and Florey, 2018; Durgan et al., 2017; Overholtzer et al., 2007). Key molecules for this process are β-catenin, E-cadherin, and Rho-associated coiled coil-containing protein kinase (ROCK) (Overholtzer et al., 2007). It has also been shown that α-catenin is important for entosis in various cancer cell lines (Wang et al., 2015). To determine whether live T cell capture by hepatic epithelia is similar to entosis, β-catenin and E-cadherin were detected by specific antibody staining in co-cultures using confocal microscopy. We noted evidence of entosis in Huh-7 cells engulfing neighboring cells in E-cadherin-rich vesicles; therefore, the cells were capable of generating E-cadherin-rich entotic vesicles in our model (Figure 4A). However, β-catenin, but not E-cadherin, associated with the T cell-containing vesicle during enclysis (Figures 4B and 4C; Figure S4A). Of note, there was no association of β-catenin with efferosomes containing necrotic T cells (Figure S4B).

To investigate the role of adherens junctions in enclysis, we imaged α- and β- catenin in enclysis intermediates, as for entosis (Overholtzer et al., 2007; Wang et al., 2015). Unlike β-catenin, there was no association of α-catenin at the site of T cell capture or the enclytic vesicle in enclysis intermediates (Figures S4 and S5). β-Catenin$^+$ areas on the enclytic vesicle were also observed in hepatocytes in human liver explants from patients with chronic liver disease (Figure 4D; Figure S6A), although the vesicles were not always homogenously positive for β-catenin. The lack of β-catenin$^+$ association with T cells in the hepatic sinusoids is shown for comparison (Figure S6B).

Live CD4$^+$ T cell capture was not perturbed by the suicidal emperipolesis inhibitor wortmannin or the entosis inhibitors for ROCK Y-27632 and H-1152 but was reduced when hepatic cells were treated with the actin polymerization inhibitors latrunculin A and cytochalasin D and the myosin II inhibitor blebbistatin (Figures 4E–4G). Engulfment of live CD4$^+$ T cells by hepatocytes is therefore mechanistically distinct from entosis and suicidal emperipolesis.

Most Enclytic Vesicles Containing Activated CD4$^+$ T Cells Did Not Acidify over Time

Capture of necrotic cells results in formation of an acidifying phagosome that fuses with lysosomes delivering digestive enzymes, which leads to degradation of the cell debris (Levin et al., 2016). Phagosome acidification is mediated by proton pumps (Lukacs et al., 1990) and can be imaged using engulfed dextran conjugated to pHrodo Red, a colorless dye that fluoresces brightly as the pH level drops. This sensitive pH indicator was added to the cell culture medium to allow concomitant internalization with capture of live or necrotic CD4$^+$ T cells. T cells were stimulated with anti-CD3+anti-CD28/interleukin-2 (IL-2) to mimic activated T cells capable of crossing the sinusoidal endothelia to reach hepatocytes in the liver. Complete cell

internalization was confirmed 5 h later by CellMask exclusion. Although hepatocytes were able to acidify phagosomes containing necrotic cells, vesicles containing live T cells most frequently did not tend to acidify (Figure 5A). Of note, time-lapse experiments indicated that efferosomes acidify rapidly following engulfment, but less than 20% of enclytic vesicles were positive for pHrodo Red (Figure 5B).

We were interested to assess whether hepatocytes were able to process live lymphocytes *in vitro*. Wang et al. (2009) demonstrated that various epithelial cancer cell lines engulf live natural killer (NK) cells in a process that involves E-cadherin junctions and leads to rapid deletion of the NK cells in intracellular compartments via programmed cell death (Qian and Shi, 2009). To determine whether Huh-7 cells engulf T cells in a similar process, we added primary CD4$^+$ T cells, Jurkat cells, NKL cells, or Raji B cells to Huh-7 cells overnight and then measured the frequency of cell-in-cell structures (Figure 5C). We also assessed the association of the lysosomal membrane protein LAMP-1 with vesicles containing live, apoptotic, and necrotic cells (Figures 5D and 5E). The use of T cell, NK cell, and B cell lines reduced the natural heterogeneity expected in primary cells and prevented any potential cytolytic effects. Hepatocyte tumor cells (Huh-7) were able to internalize live NKL cells as reported for other epithelial tumor cells (Wang et al., 2009) to the same frequency as T cells, but the B cell line Raji was not engulfed, demonstrating selectivity. Apoptotic and necrotic preparations of these cells were captured effectively. However, live primary and Jurkat T cells found in enclytic vesicles did not associate with LAMP-1$^+$ vesicles. NKL cells (often internalized) and Raji B cells (rarely internalized, likely apoptotic) were found in efferosomes associating with LAMP-1$^+$ vesicles. These experiments demonstrate that Huh-7 cells were capable of deleting live NKL cells in lysosomal compartments, but this was not the outcome for engulfed CD4$^+$ T cells.

T Cell-Containing Vesicles in Hepatocytes Were Connected to the Endocytic Pathway

We were interested in understanding the prolonged viability of T cells confined in hepatocytes *in vitro* during enclysis; in time-lapse imaging experiments at 37°C, we noted progressive endocytosis of CellMask-labeled plasma membranes and trafficking of endosomes to T cell-containing vesicles (Figure S7A). In 10 min following CellMask addition, endosomes were found to bind the enclytic vesicle. By 60 min, multiple membrane-derived vesicles associated with the T cell-containing vesicle, leading to fusion with the vesicle membrane. CellMask Membrane binds undefined proteins on the plasma membrane. Conversely, FM1-43 is a lipophilic styryl dye labeling membrane compartments that internalize rapidly. We studied FM1-43$^+$ endosomes budding from the plasma membrane in cells

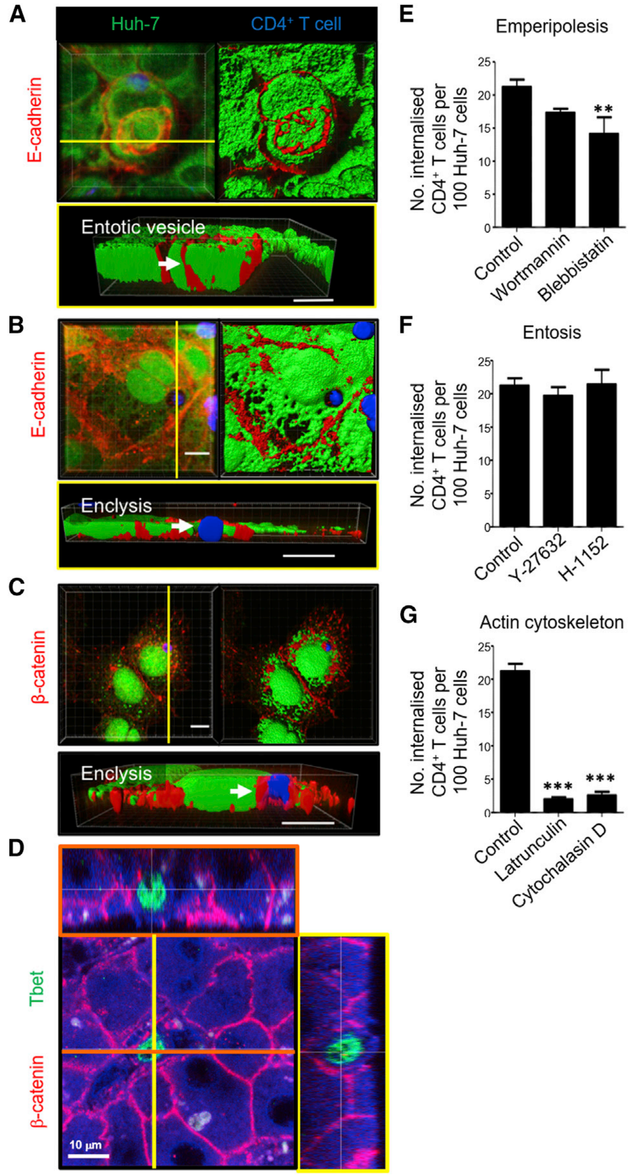

Figure 4. Enclysis Forms β-Catenin-Rich Vesicles and Is Distinct from Entosis and Suicidal Emperipolesis

(A) Huh-7 cells (CMFDA, green) were co-cultured with Jurkat T cells (BMQC, blue), fixed, stained for anti-E-cadherin (anti-mouse Alexa Fluor 594, red), and imaged by confocal microscopy. The arrow indicates an E-cadherin$^+$ entotic vesicle containing another Huh-7 cell.

(B) A binucleate Huh-7 cell containing a T cell in a vesicle devoid of E-cadherin staining.

(C) Huh-7 cell containing a T cell in a β-catenin$^+$ enclytic vesicle.

(D) Orthogonal confocal image of a 30-μm-thick section from formalin-fixed, paraffin-embedded tissue from a cirrhotic liver explant stained for β-catenin and the CD4$^+$ T cell transcription factor Tbet. Immunohistochemistry reveals autofluorescence, which helps visualize hepatocyte cytoplasm (blue) and,typical for the liver, cell debris that autofluoresces in all channels (white).

(E) Pre-treatment of Huh-7 cells with wortmannin, which inhibits suicidal emperipolesis, did not affect enclysis. However, the myosin-II and macropinocytosis inhibitor blebbistatin (50 μM) reduced T cell capture, as measured by confocal microscopy.

(F) Entosis inhibitor treatment of Huh-7 cells with the ROCK inhibitors Y-27632 and H1152 (10 μM) did not affect enclysis.

(G) Enclysis was perturbed by Huh-7 pre-treatment with the actin organization inhibitors latrunculin A (0.1 μM) and cytochalasin D (1 μM).

Data were derived from three independent experiments. $^{**}p < 0.01$, $^{***}p < 0.001$, Student's non-parametric paired t test (Wilcoxon). Scale bars represent 10 μm.

See also Figures S4–S6.

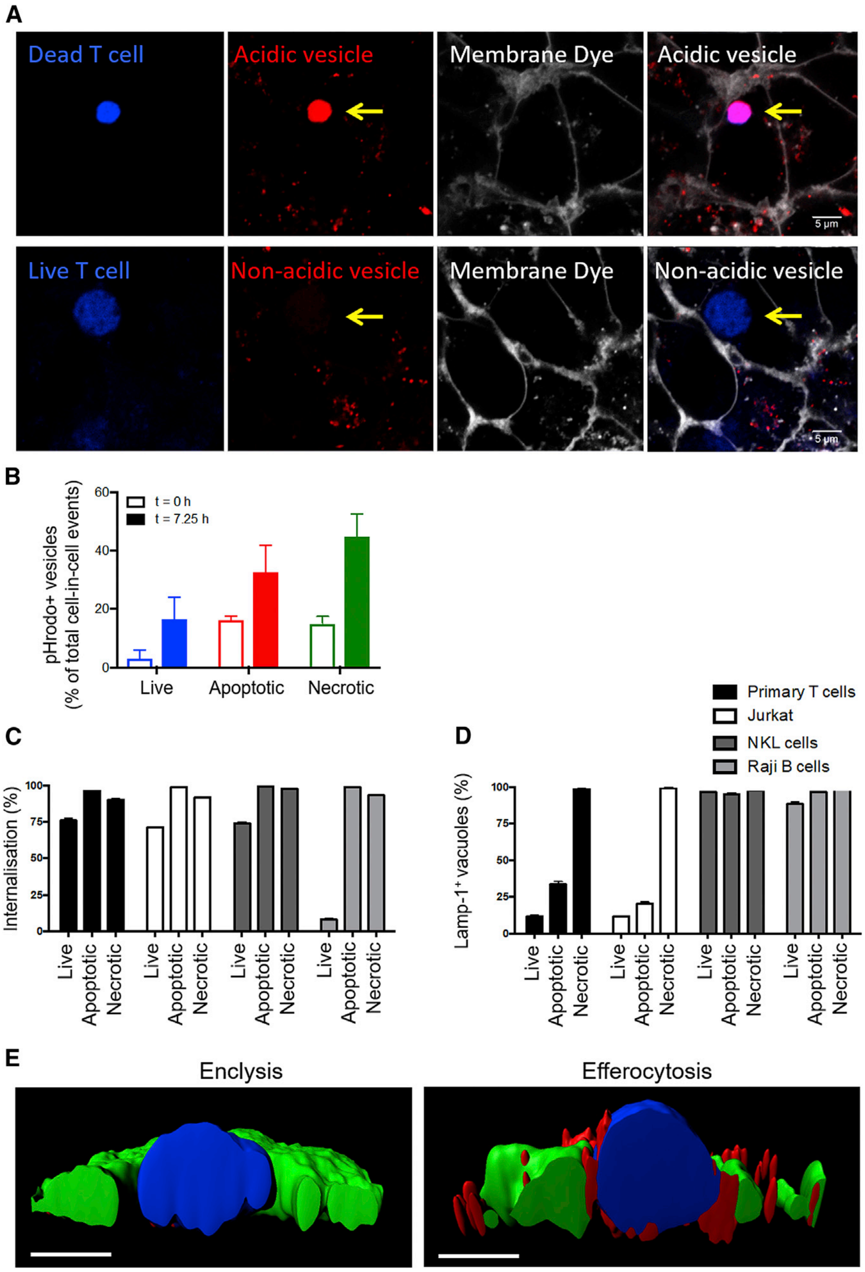

Figure 5. Enclytic Vesicles Containing Live Stimulated CD4⁺ T Cells Did Not Acidify and Did Not Associate with LAMP-1⁺ Lysosomes

(A) Huh-7 cells were co-cultured with necrotic (heat-killed) or live peripheral blood-derived $CD4^+$ T cells activated via CD3+CD28/IL-2 (BMQC, blue) in the presence of pHrodo Red dextran in the culture medium (colorless, fluoresces red in acidic compartments). Complete internalization of T cells was confirmed by CellMask Plasma Membrane Deep Red addition to the medium, which labels all exposed cell membranes (white). Acidic endosomes arising from the membrane are seen in red in Huh-7 cells. The arrows indicate a phagosome containing a necrotic T cell and an enclytic vesicle containing a live T cell.

(B) $CD4^+$ T cells were isolated from peripheral blood and co-cultured overnight with Huh-7 cells in the presence of pHrodo Red dextran. Live (blue), apoptotic (staurosporine-treated, red), and necrotic (heat-killed, green) cells were imaged every 15 min on a CQ1 high-content benchtop microscope, and the proportion of pHrodo Red^+ vesicles was quantified at t = 0 (open bars) and t = 7.25 h (closed bars). Data shown are mean ± SD from triplicate wells (three fields of view) from one experiment.

(C and D) Live, apoptotic (staurosporine-treated), or necrotic (heat-killed) lymphocytes were labeled (BMQC, blue) and co-cultured with Huh-7 cells (CMFDA, green) for 24 h. The cells were fixed and stained with anti-LAMP-1 (anti-mouse Alexa 594). Cell-in-cell structures (C) and LAMP-1^+ vesicles (D) were enumerated by confocal microscopy.

(E) IMARIS rendering of cross-sections of vesicles on Huh-7 cells (CMFDA, green) containing live or heat-killed T cells (BMQC, blue), with LAMP-1^+ vesicles shown in red. Scale bars indicate 5 µm.

See also Figure S7.

previously stained with CellMask dye to pinpoint internalized T cells (Figure S7B). FM1-43^+ endosomes fused with the T cell-containing vesicle membrane in as little as 2 min and continued to accumulate particularly at preferred "docking sites." To our knowledge, this has not been described previously for any type of cell-in-cell structure.

It is possible that nutrients from the cell culture medium continue to be delivered to the enclytic vesicle by endocytosis. To test this hypothesis, we used fluorescein isothiocyanate (FITC)-labeled dextran in the cell culture medium, which accumulated in the T cell-containing vesicle following overnight incubation (Figure S7C). The enclytic vesicle is therefore connected to the endocytic pathway.

Hepatocytes Preferentially Engulfed Regulatory T Cells Compared with Non-Treg Cells, and Treg Cell-Containing Vesicles Acidified Overnight

$CD4^+$ T cells comprise a diverse population of cells with the potential to drive an immune response (usually $Tbet^+$ T helper 1-type cells in the liver) or to dampen T cell proliferation (Treg cells). Treg cells usually comprise 5%–10% of peripheral blood $CD4^+$ T cells. We noticed that, following primary T cell internalization in the absence of *in vitro* stimulation, over 60% of internalized T cells were positive for the Treg cell-linked transcription factor Foxp3 (Figure 6A). Foxp3 in mice is restricted to Treg cells, but in humans it can also emerge following stimulation (Allan et al., 2007; Wang et al., 2007). We therefore isolated Treg cells using magnetic beads coupled to antibodies against cell surface receptors ($CD4^+CD127^{low}CD49^-$ Treg cells, STEMCELL Technologies). A custom kit was generated to isolate $CD4^+CD25^-$ non-Treg cells. 500,000 T cells were added to a confluent monolayer of Huh-7

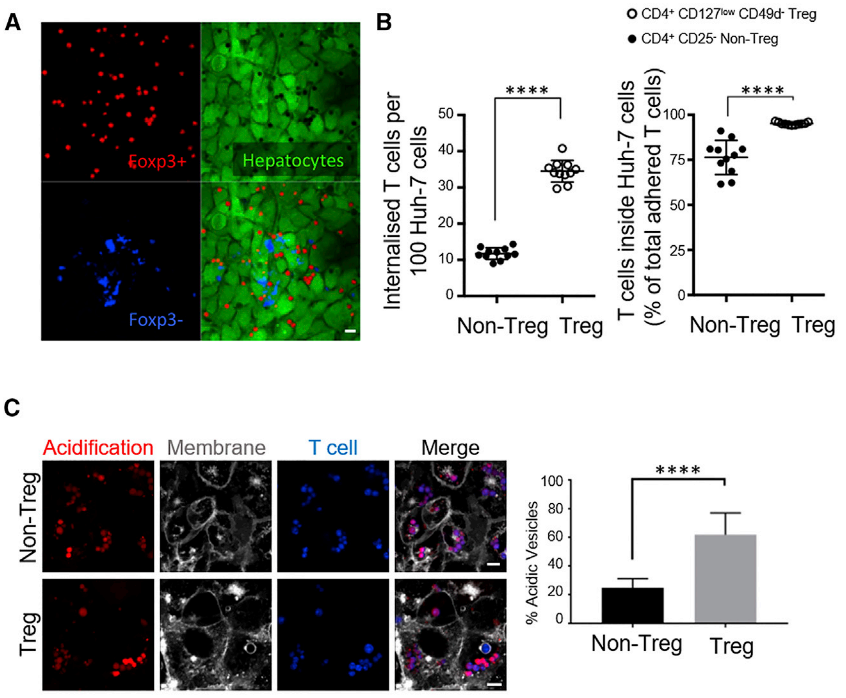

Figure 6. Hepatocytes Captured Treg Cells More Efficiently than Non-Treg Cells and Selectively Acidified Treg Cell-Containing Vesicles

(A) $CD4^+$ T cells were isolated from peripheral blood (BMQC, blue) and co-cultured overnight with a confluent monolayer of Huh-7 cells (CMFDA, green). The cells were fixed and stained for Foxp3 to detect T regulatory (Treg) cells (red, anti-mouse immunoglobulin G [IgG]-Alexa 594). Approximately 60% of captured T cells were $Foxp3^+$. $Foxp3^-$ T cells were labeled more brightly with BMQC and easy to distinguish.

(B) Treg and non-Treg cells were isolated from peripheral blood using magnetic beads and added to wells containing Huh-7 cells at equal numbers. 3 h later, non-associating T cells were washed off, and internalized cells were enumerated by confocal microscopy. Each dot represents average internalized cells per 100 Huh-7 cells in independent experiments.

(C) Treg and non-Treg cells were isolated from peripheral blood and co-cultured overnight with Huh-7 cells in the presence of colorless pHrodo Red dextran, which fluoresces under low-pH conditions. Bright acidic vesicles were enumerated in random fields of view and plotted for three independent experiments.

Error bars show SD. ****$p < 0.0001$. Statistics were derived from an unpaired, two-tailed Student's t test.

cells in a 24-well plate and incubated for 3 h, and non-associated cells were washed off. Hepatocytes were over three times more efficient at capturing Treg cells compared with non-Treg cells (Figure 6B). Overall, the total percentage of internalized Treg cells was higher (~98%) compared with the percentage of internalized non-Treg cells (~75%). Huh-7 cells are therefore more efficient at engulfing Treg cells compared with non-Treg cells.

We next set out to compare the outcome of internalized Treg and non-Treg cells using the pH indicator dye pHrodo Red (Figure 6C). Over 60% of Treg cells found in Huh-7 cells resided in acidic vesicles following overnight co-culture. Only about 20% of engulfed non-Treg cells were detected in bright red acidic vesicles. These experiments show that the outcome of enclysis may be tailored to the T cell subset.

$Foxp3^+$ T Cells Were Found in Hepatocytes More Frequently than $Tbet^+$ T Cells *In Vivo*

To determine whether Treg cells were found inside hepatocytes more frequently *in vivo*, we enumerated $Foxp3^+$ cells (mostly Treg cells) and $Tbet^+$ cells (mostly Th1) in hepatocytes in end-stage disease liver explants from 80 patients with various disease etiologies (Figure 7A). Patients were stratified according to condition, including donor livers rejected for transplantation (non-cirrhotic), autoimmune family disorders (autoimmune hepatitis, primary biliary cholangitis, and primary sclerosing cholangitis), metabolic injuries (alcohol or fatty liver diseases), and chronic viral infection (hepatitis B or C) (Table S1). Overall, more $Tbet^+$ cells were detected in the liver parenchyma compared with $Foxp3^+$ cells, as expected (Figure 7B). However, under all conditions tested, most of the $Foxp3^+$ cells that were found within the parenchyma were in hepatocytes (Figure 7C). The frequency of $Tbet^+$ cells in hepatocytes was consistently less than 50% of the total parenchymal population. Therefore, *in vivo*, the frequency of $Foxp3^+$ cells in hepatocytes was higher compared with $Tbet^+$ cells, suggesting that enclysis favors Treg cells *in vivo* as well as *in vitro*.

DISCUSSION

The liver has long been suggested as a graveyard for spent immune cells, which is particularly important in chronic inflammatory conditions (Crispe et al., 2000). The contribution of hepatocytes to extra-thymic immune tolerance by deletion of autoreactive cytotoxic $CD8^+$ cells highlights an active role of hepatocytes in immune regulation (Benseler et al., 2011; Sierro et al., 2015). The study describing suicidal emperipolesis demonstrated rapid engulfment of autoreactive cytotoxic cells recognizing their cognate antigen on hepatocytes; enclysis did not depend on antigen recognition. Further, unlike reported for $CD8^+$ cells, $CD4^+$ T helper cells did not show peripatetic behavior in hepatocytes and remained rounded despite mitochondrial activity (Figure 1; Figure S1; Videos S1, S2, S3, and S4).

Of note, early T cell progenitors (thymocytes) have also been reported to form cell-in-cell structures when completely engulfed by thymic nurse cells in the thymus cortex (Guyden et al., 2015; Li et al., 1992). This process is thought to nurture immature $\alpha\beta TCR^{low}CD4^+CD8^+$ thymocytes and release selected thymocytes that develop a mature phenotype. The precise mechanism of thymocyte capture is unknown, and there are no tools to modulate this process; therefore, its biological effect

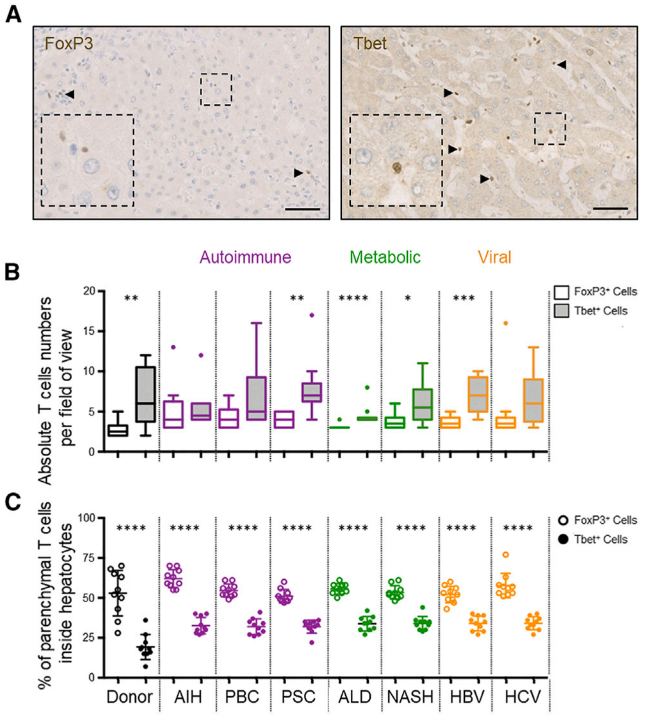

Figure 7. Foxp3⁺ Cells Were Found in Hepatocytes More Frequently Than Tbet⁺ Cells in Donor Livers and in Explants from Patients with Chronic Liver Disease

(A) Immunohistochemistry images of Foxp3$^+$ and Tbet$^+$ cells (DAB, brown) in livers from a patient with autoimmune hepatitis (AIH).

(B and C) Absolute numbers (B) and frequencies (C) of Foxp3$^+$ or Tbet$^+$ CD4$^+$ T cells in hepatocytes in immunohistochemistry-stained sections from 80 patients with end-stage liver disease. Manual counts were performed by two independent scientists. PBC, primary biliary cholangitis; PSC, primary sclerosing cholangitis; ALD, alcoholic liver disease; NASH, non-alcoholic steatohepatitis; HBV, hepatitis B virus infection; HCV, hepatitis C virus infection. *$p < 0.05$, **$p < 0.01$, ***$p < 0.001$, ****$p < 0.0001$, Student's non-parametric paired t test (Wilcoxon).

on T cell development remains elusive. Similarly, hepatocytes may act as nurse cells to promote maturation of erythrocytes, as shown in frogs (Barni and Bernocchi, 1991) and in human fetal liver Kupffer cells (Lee et al., 1999). In these contexts, the host cells can support the survival of engulfed cells using mechanisms that are poorly defined.

The biological role of CD4$^+$ T helper cells is to guide immune responses of other cell types, a function critical for initiation and resolution of inflammatory responses tailored to the cause of injury. A characteristic example observed in chimpanzees is the failure of cytotoxic cells to control hepatitis C infection in the absence of CD4$^+$ T cells (Grakoui et al., 2003). T cell migration to the liver parenchyma that leads to their engulfment by hepatocytes may restrict the abundance of T helper cell cytokines and perturb immune regulation. T cell plasticity gives rise to regulatory and effector subsets with different immunomodulatory cytokine expression profiles. In the present study, hepatocytes displayed different modes of processing Treg and non-Treg cells both in the frequency of capture and during processing of the T cell-containing vesicle.

ICAM-1 played a role in early adhesion of T cells to hepatocytes, and ICAM-1 inhibition reduced cell-in-cell events, but ICAM-1 blockade had no effect on T cell binding to hepatocytes beyond 15 min. E-cadherin and β-catenin are molecules important for epithelial cell-in-cell structures (entosis) (Overholtzer et al., 2007), but E-cadherin did not associate with the T cell containing-vesicle in hepatocytes. The enclytic vesicle was rich in β-catenin, which enabled detection of enclysis in human liver tissue sections. The precise molecular parameters of T cell-hepatocyte interactions require further investigation.

We disproved the hypothesis that hepatocytes capture CD4$^+$ T cells via efferocytosis; enclysis involved cellular blebs and lamellipodia instead of ruffles typical of phagocytic engulfment, had a slower kinetics profile, and led to intracellular T cell-containing vesicles that did not acidify following overnight incubation. Engulfment of large amounts of fluid is known as macropinocytosis, and this term encompasses a wide range of cellular capture mechanisms that allow internalization of macromolecules, viruses, and pathogens (Mercer and Helenius, 2009; Swanson and Watts, 1995). Enclysis shares features with macropinocytosis because it depends on the actin cytoskeleton and generates a large vesicle concomitant with fluid uptake from the extracellular space, which separates from the host cell membrane and traffics inside the cell. However, the two processes are fundamentally different because macropinocytosis drives non-specific engulfment of fluids, whereas T cell capture by hepatocytes was restricted to CD4$^+$ T cells and was most efficient for Treg cells. Further, macropinocytosis triggering is rapid and involves global actin cytoskeletal activation (Steinman et al., 1976; Swanson, 1989), whereas T cell internalization into hepatocytes was slow and initiated membrane lamellipodia or blebs proximal to the T cell-associating region.

To understand the mode of T cell survival in hepatocytes (non-Treg cells), our experiments showed that the T cell-containing vesicles were connected to the endocytic pathway. It is tempting to speculate regarding the biological role of T cell engulfment by hepatocytes in the absence of killing. CD4$^+$ T cells license cytotoxic cell function by priming antigen-presenting cells; removing these during liver antigen recognition by hepatocyte internalization may contribute to tolerance induction (Crispe, 2016; Grakoui and Crispe, 2016; Smith et al., 2004).

From a cell biology perspective, there is little in the literature describing hepatocyte intracellular vesicles and their communication with the extracellular space. Malaria parasites invade hepatocyte cytoplasm in a manner dependent on Kupffer cells, which leads to generation of parasitophorous vacuoles expressing parasite-encoded proteins (Baer et al., 2007; Pradel and Frevert, 2001). Parasites can interact with hepatocytes through this vacuolar membrane and contribute to host cell membrane reorganization of transmembrane domain-containing proteins, palmitoylated and myristoylated proteins, and glycosylphosphatidylinositol-anchored proteins (Burda et al., 2017; Schnider et al., 2018). We demonstrated continuous cumulating endosome fusion with the T cell-containing vesicle using membrane dyes and documented delivery of fluorescent dextran from the extracellular space to the enclytic vesicle. The precise composition of this vesicle membrane remains to be established.

Taken together, our experiments define a new mode of lymphocyte-hepatocyte interaction and characterize the phenomenon of enclysis, which gives rise to opportunities for new studies to understand CD4$^+$ T cell dynamics in the liver.

STAR★METHODS

Detailed methods are provided in the online version of this paper and include the following:

- KEY RESOURCES TABLE
- LEAD CONTACT AND MATERIALS AVAILABILITY
- EXPERIMENTAL MODEL AND SUBJECT DETAILS
  - Human liver tissue
  - Primary cell isolation
  - Cell lines
- METHOD DETAILS
  - Paraffin-embedding of tissue
  - Immunohistochemistry
  - Co-culture assays
  - Cell treatments and induction of cell death
  - Cell-in-cell assays
  - Live cell imaging
  - Immunofluorescence
  - Scanning electron microscopy
  - Time-lapse microscopy using the CQ1 High-Content Benchtop Microscope
  - Flow Cytometry
- QUANTIFICATION AND STATISTICAL ANALYSIS
  - Graphical Abstract
- DATA AND CODE AVAILABILITY

SUPPLEMENTAL INFORMATION

Supplemental Information can be found online at https://doi.org/10.1016/j.celrep.2019.09.068.

ACKNOWLEDGMENTS

We are grateful to patients and clinicians at the Queen Elizabeth Hospital Birmingham for generous access to blood and tissue; Dr. Louisa Jefferies (University of Birmingham, UK) for advice regarding custom non-Treg cell isolation; Prof. Jane McKeating (University of Oxford, UK), Prof. David C. Wraith (University of Birmingham, UK), and Prof. Harvey McMahon (MRC Laboratory of Molecular Biology, UK) for reagents and helpful discussions; Dr. Rob Shaw for support with Birmingham Technology Hub imaging resources; Ms. Uzba Naheed, Mr. Paul Stanley, and Mrs. Theresa Morris (School of Metallurgy and Materials) for assistance with scanning electron microscopy; Dr. Christopher Weston, Mrs. Rhianne Hutchinson-Davies, and Dr. Richard Singleton for digital image processing (University of Birmingham, UK); and Ms. Janine Fear for expert administration at the Centre for Liver and Gastrointestinal research (University of Birmingham, UK). S.P.D. was funded by a PhD studentship from the Medical Research Council (MRC) Centre for Immune Regulation. X.L. was funded by a Guangzhou Municipal Government (GMG) award (2016201604030021 to Y.H. and Z.S.). A.L.W. was funded by a Wellcome Trust PhD studentship in Mechanisms of Inflammatory Diseases. Y.S.L. was funded by an MRC Confidence in Concept Award (to Z.S.). This work was initiated thanks to support from a small seed award by the Wellcome Trust Institutional Support Fund (to Z.S.). Z.S. has subsequently received funding from the MRC, Wellcome Trust, GMG, The Birmingham Children's Hospital Research Foundation, and a Royal Society Dorothy Hodgkin fellowship. J.G. is the recipient of a Sir Henry Dale Fellowship from the Wellcome Trust and the Royal Society (107653/Z/15/Z). R.C.M. is supported by the BBSRC, an ERC consolidator award (MitoFun), and a Wolfson Research Merit Award from the Royal Society.

AUTHOR CONTRIBUTIONS

Conceptualization, Z.S., O.Q., G.M.R., D.H.A., R.C.M., and S.G.H.; Methodology, Z.S., O.Q., and G.M.R.; Investigation, Z.S., O.Q., G.M.R., S.P.D., J.G., R.R., M.L., X.L., R.G., Y.S.L., E.B., and A.L.W.; Writing – Original Draft, Z.S. and S.P.D.; Writing – Review & Editing, Z.S., S.P.D., O.Q., A.L.W., Y.H., X.L., and S.G.H.; Funding Acquisition, Z.S. and Y.H.; Resources, Z.S., O.Q., N.M.B., G.M.R., and Y.H.; Supervision, Z.S., O.Q., G.M.R., and Y.H.

DECLARATION OF INTERESTS

The authors declare no competing interests.

Received: February 5, 2019
Revised: June 3, 2019
Accepted: September 20, 2019
Published: November 5, 2019

REFERENCES

Allan, S.E., Crome, S.Q., Crellin, N.K., Passerini, L., Steiner, T.S., Bacchetta, R., Roncarolo, M.G., and Levings, M.K. (2007). Activation-induced FOXP3 in human T effector cells does not suppress proliferation or cytokine production. Int. Immunol. *19*, 345–354.

Baer, K., Roosevelt, M., Clarkson, A.B., Jr., van Rooijen, N., Schnieder, T., and Frevert, U. (2007). Kupffer cells are obligatory for Plasmodium yoelii sporozoite infection of the liver. Cell. Microbiol. *9*, 397–412.

Barni, S., and Bernocchi, G. (1991). Internalization of erythrocytes into liver parenchymal cells in naturally hibernating frogs (Rana esculenta L.). J. Exp. Zool. *258*, 143–150.

Benseler, V., Warren, A., Vo, M., Holz, L.E., Tay, S.S., Le Couteur, D.G., Breen, E., Allison, A.C., van Rooijen, N., McGuffog, C., et al. (2011). Hepatocyte entry leads to degradation of autoreactive CD8 T cells. Proc. Natl. Acad. Sci. USA *108*, 16735–16740.

Bertolino, P., Trescol-Biémont, M.C., and Rabourdin-Combe, C. (1998). Hepatocytes induce functional activation of naive CD8+ T lymphocytes but fail to promote survival. Eur. J. Immunol. *28*, 221–236.

Burda, P.C., Caldelari, R., and Heussler, V.T. (2017). Manipulation of the Host Cell Membrane during *Plasmodium* Liver Stage Egress. MBio *8*, e00139-17.

Burghardt, S., Erhardt, A., Claass, B., Huber, S., Adler, G., Jacobs, T., Chalaris, A., Schmidt-Arras, D., Rose-John, S., Karimi, K., and Tiegs, G. (2013). Hepatocytes contribute to immune regulation in the liver by activation of the Notch signaling pathway in T cells. J. Immunol. *191*, 5574–5582.

Burghardt, S., Claass, B., Erhardt, A., Karimi, K., and Tiegs, G. (2014). Hepatocytes induce Foxp3$^+$ regulatory T cells by Notch signaling. J. Leukoc. Biol. *96*, 571–577.

Calne, R.Y., Sells, R.A., Pena, J.R., Davis, D.R., Millard, P.R., Herbertson, B.M., Binns, R.M., and Davies, D.A. (1969). Induction of immunological tolerance by porcine liver allografts. Nature *223*, 472–476.

Crispe, I.N. (2016). Hepatocytes as Immunological Agents. J. Immunol. *196*, 17–21.

Crispe, I.N., Dao, T., Klugewitz, K., Mehal, W.Z., and Metz, D.P. (2000). The liver as a site of T-cell apoptosis: graveyard, or killing field? Immunol. Rev. *174*, 47–62.

Davies, S.P., Reynolds, G.M., and Stamataki, Z. (2018). Clearance of Apoptotic Cells by Tissue Epithelia: A Putative Role for Hepatocytes in Liver Efferocytosis. Front. Immunol. *9*, 44.

Durgan, J., and Florey, O. (2018). Cancer cell cannibalism: Multiple triggers emerge for entosis. Biochim Biophys Acta Mol Cell Res *1865*, 831–841.

Durgan, J., Tseng, Y.Y., Hamann, J.C., Domart, M.C., Collinson, L., Hall, A., Overholtzer, M., and Florey, O. (2017). Mitosis can drive cell cannibalism through entosis. eLife *6*, e27134.

Grakoui, A., and Crispe, I.N. (2016). Presentation of hepatocellular antigens. Cell. Mol. Immunol. *13*, 293–300.

Grakoui, A., Shoukry, N.H., Woollard, D.J., Han, J.H., Hanson, H.L., Ghrayeb, J., Murthy, K.K., Rice, C.M., and Walker, C.M. (2003). HCV persistence and immune evasion in the absence of memory T cell help. Science *302*, 659–662.

Grant, C.R., and Liberal, R. (2017). Liver immunology: How to reconcile tolerance with autoimmunity. Clin. Res. Hepatol. Gastroenterol. *41*, 6–16.

Guidotti, L.G., Inverso, D., Sironi, L., Di Lucia, P., Fioravanti, J., Ganzer, L., Fiocchi, A., Vacca, M., Aiolfi, R., Sammicheli, S., et al. (2015). Immunosurveillance of the liver by intravascular effector CD8(+) T cells. Cell *161*, 486–500.

Guyden, J.C., Martinez, M., Chilukuri, R.V., Reid, V., Kelly, F., and Samms, M.O. (2015). Thymic Nurse Cells Participate in Heterotypic Internalization and Repertoire Selection of Immature Thymocytes; Their Removal from the Thymus of Autoimmune Animals May be Important to Disease Etiology. Curr. Mol. Med. *15*, 828–835.

Heymann, F., and Tacke, F. (2016). Immunology in the liver–from homeostasis to disease. Nat. Rev. Gastroenterol. Hepatol. *13*, 88–110.

Lee, W.B., Erm, S.K., Kim, K.Y., and Becker, R.P. (1999). Emperipolesis of erythroblasts within Kupffer cells during hepatic hemopoiesis in human fetus. Anat. Rec. *256*, 158–164.

Levin, R., Grinstein, S., and Canton, J. (2016). The life cycle of phagosomes: formation, maturation, and resolution. Immunol. Rev. *273*, 156–179.

Levitsky, J., and Feng, S. (2018). Tolerance in clinical liver transplantation. Hum. Immunol. *79*, 283–287.

Li, Y., Pezzano, M., Philp, D., Reid, V., and Guyden, J. (1992). Thymic nurse cells exclusively bind and internalize CD4+CD8+ thymocytes. Cell. Immunol. *140*, 495–506.

Lukacs, G.L., Rotstein, O.D., and Grinstein, S. (1990). Phagosomal acidification is mediated by a vacuolar-type H(+)-ATPase in murine macrophages. J. Biol. Chem. *265*, 21099–21107.

Mee, C.J., Harris, H.J., Farquhar, M.J., Wilson, G., Reynolds, G., Davis, C., van IJzendoorn, S.C., Balfe, P., and McKeating, J.A. (2009). Polarization restricts hepatitis C virus entry into HepG2 hepatoma cells. J. Virol. *83*, 6211–6221.

Mercer, J., and Helenius, A. (2009). Virus entry by macropinocytosis. Nat. Cell Biol. *11*, 510–520.

Nakaya, M., Tanaka, M., Okabe, Y., Hanayama, R., and Nagata, S. (2006). Opposite effects of rho family GTPases on engulfment of apoptotic cells by macrophages. J. Biol. Chem. *281*, 8836–8842.

Oo, Y.H., Weston, C.J., Lalor, P.F., Curbishley, S.M., Withers, D.R., Reynolds, G.M., Shetty, S., Harki, J., Shaw, J.C., Eksteen, B., et al. (2010). Distinct roles for CCR4 and CXCR3 in the recruitment and positioning of regulatory T cells in the inflamed human liver. J. Immunol. *184*, 2886–2898.

Overholtzer, M., and Brugge, J.S. (2008). The cell biology of cell-in-cell structures. Nat. Rev. Mol. Cell Biol. *9*, 796–809.

Overholtzer, M., Mailleux, A.A., Mouneimne, G., Normand, G., Schnitt, S.J., King, R.W., Cibas, E.S., and Brugge, J.S. (2007). A nonapoptotic cell death process, entosis, that occurs by cell-in-cell invasion. Cell *131*, 966–979.

Pichard, L., Raulet, E., Fabre, G., Ferrini, J.B., Ourlin, J.C., and Maurel, P. (2006). Human hepatocyte culture. Methods Mol. Biol. *320*, 283–293.

Pradel, G., and Frevert, U. (2001). Malaria sporozoites actively enter and pass through rat Kupffer cells prior to hepatocyte invasion. Hepatology *33*, 1154–1165.

Qian, Y., and Shi, Y. (2009). Natural killer cells go inside: entosis versus cannibalism. Cell Res. *19*, 1320–1321.

Qian, S., Wang, Z., Lee, Y., Chiang, Y., Bonham, C., Fung, J., and Lu, L. (2001). Hepatocyte-induced apoptosis of activated T cells, a mechanism of liver transplant tolerance, is related to the expression of ICAM-1 and hepatic lectin. Transplant. Proc. *33*, 226.

Robertson, M.J., Cochran, K.J., Cameron, C., Le, J.M., Tantravahi, R., and Ritz, J. (1996). Characterization of a cell line, NKL, derived from an aggressive human natural killer cell leukemia. Exp. Hematol. *24*, 406–415.

Sana, G., Lombard, C., Vosters, O., Jazouli, N., Andre, F., Stephenne, X., Smets, F., Najimi, M., and Sokal, E.M. (2014). Adult human hepatocytes promote CD4(+) T-cell hyporesponsiveness via interleukin-10-producing allogeneic dendritic cells. Cell Transplant. *23*, 1127–1142.

Schnider, C.B., Bausch-Fluck, D., Brühlmann, F., Heussler, V.T., and Burda, P.C. (2018). BioID Reveals Novel Proteins of the *Plasmodium* Parasitophorous Vacuole Membrane. MSphere *3*, e00522-17.

Shetty, S., Weston, C.J., Oo, Y.H., Westerlund, N., Stamataki, Z., Youster, J., Hubscher, S.G., Salmi, M., Jalkanen, S., Lalor, P.F., and Adams, D.H. (2011). Common lymphatic endothelial and vascular endothelial receptor-1 mediates the transmigration of regulatory T cells across human hepatic sinusoidal endothelium. J. Immunol. *186*, 4147–4155.

Shetty, S., Lalor, P.F., and Adams, D.H. (2018). Liver sinusoidal endothelial cells - gatekeepers of hepatic immunity. Nat. Rev. Gastroenterol. Hepatol. *15*, 555–567.

Sierro, F., Tay, S.S., Warren, A., Le Couteur, D.G., McCaughan, G.W., Bowen, D.G., and Bertolino, P. (2015). Suicidal emperipolesis: a process leading to cell-in-cell structures, T cell clearance and immune homeostasis. Curr. Mol. Med. *15*, 819–827.

Smith, C.M., Wilson, N.S., Waithman, J., Villadangos, J.A., Carbone, F.R., Heath, W.R., and Belz, G.T. (2004). Cognate CD4(+) T cell licensing of dendritic cells in CD8(+) T cell immunity. Nat. Immunol. *5*, 1143–1148.

Steinman, R.M., Brodie, S.E., and Cohn, Z.A. (1976). Membrane flow during pinocytosis. A stereologic analysis. J. Cell Biol. *68*, 665–687.

Swanson, J.A. (1989). Phorbol esters stimulate macropinocytosis and solute flow through macrophages. J. Cell Sci. *94*, 135–142.

Swanson, J.A. (2008). Shaping cups into phagosomes and macropinosomes. Nat. Rev. Mol. Cell Biol. *9*, 639–649.

Swanson, J.A., and Watts, C. (1995). Macropinocytosis. Trends Cell Biol. *5*, 424–428.

Wang, J., Ioan-Facsinay, A., van der Voort, E.I., Huizinga, T.W., and Toes, R.E. (2007). Transient expression of FOXP3 in human activated nonregulatory CD4+ T cells. Eur. J. Immunol. *37*, 129–138.

Wang, S., Guo, Z., Xia, P., Liu, T., Wang, J., Li, S., Sun, L., Lu, J., Wen, Q., Zhou, M., et al. (2009). Internalization of NK cells into tumor cells requires ezrin and leads to programmed cell-in-cell death. Cell Res. *19*, 1350–1362.

Wang, M., Ning, X., Chen, A., Huang, H., Ni, C., Zhou, C., Yu, K., Lan, S., Wang, Q., Li, S., et al. (2015). Impaired formation of homotypic cell-in-cell structures in human tumor cells lacking alpha-catenin expression. Sci. Rep. *5*, 12223.

Warren, A., Le Couteur, D.G., Fraser, R., Bowen, D.G., McCaughan, G.W., and Bertolino, P. (2006). T lymphocytes interact with hepatocytes through fenestrations in murine liver sinusoidal endothelial cells. Hepatology *44*, 1182–1190.

Wiegard, C., Wolint, P., Frenzel, C., Cheruti, U., Schmitt, E., Oxenius, A., Lohse, A.W., and Herkel, J. (2007). Defective T helper response of hepatocyte-stimulated CD4 T cells impairs antiviral CD8 response and viral clearance. Gastroenterology *133*, 2010–2018.

STAR★METHODS

KEY RESOURCES TABLE

REAGENT or RESOURCE	SOURCE	IDENTIFIER
Antibodies		
β-catenin antibody	Biolegend	Clone 12F7; Cat# 844601; RRID: AB_2565803
α-catenin antibody	BD Biosciences	Cat# 610193; RRID:AB_397592
FOXP3 antibody	Abcam	Cat# ab20034; RRID:AB_445284
Tbet antibody	Santa Cruz, USA	Cat# Sc-21003; RRID:AB_2200557
pan-cytokeratin antibody	Abcam	Cat# ab27988; RRID:AB_777047
CD3 antibody	Abcam	Cat# ab5690; RRID:AB_305055
CD4 antibody	Dako Agilent Pathology Solutions	Clone 4B12; Cat# M7310, RRID:AB_2728838
Anti-mouse Mouse IgG, Alexa fluor 488-conjugated	Thermo Fisher	Cat# A-11001; RRID:AB_2534069)
Anti- Mouse IgG, Alexa fluor 594-conjugated	Thermo Fisher	Cat# A-11005; RRID:AB_141372)
Anti-rabbit Mouse IgG, Alexa fluor 594-conjugated	Thermo Fisher	Cat# A-11012; RRID:AB_141359
Horseradish Peroxidase Streptavidin antibody	Vector Laboratories	Cat# SA-5004; RRID:AB_2336509)
MRP2 Antibody	Abcam	Cat# ab110740, RRID:AB_10861049
LAMP1 Antibody	BD Biosciences	Cat# 611042, RRID:AB_398355
ICAM-1 Antibody	R&D Systems	Clone BBIG –I1; Cat# BBA3, RRID:AB_356950
ICAM-2 Antibody	R&D Systems	Cat# AF244, RRID:AB_2122206
E-cadherin Antibody	BD Biosciences	Cat# 610182, RRID:AB_397581
CD44 Antibody	Abcam	Cat# ab157107
EpCAM anitbody	Biolegend	Cat# 324201, RRID:AB_756075
β2-integrin antibody	Novus biologicals	Cat# AF1730, RRID:AB_354957
β1-integrin antibody	Novus biologicals	Cat# MAB17781, RRID:AB_2129940
CD3 antibody for T cell activation	eBioscience	Clone OKT3; Cat# 16-0037-85; RRID:AB_468855
CD28 antibody for T cell activation	eBioscience	Cat# 16-0288-85, RRID:AB_468925
Biological Samples		
Explanted human tissue	Queen Elizabeth Hospital, Birmingham, UK	LREC Approval 06/Q702/61, South Birmingham, Birmingham, UK).
Whole blood in EDTA from haemochromatosis patients	Queen Elizabeth Hospital, Birmingham, UK	LREC Approval 04/Q2708/41, South Birmingham, Birmingham, UK).
Chemicals, Peptides, and Recombinant Proteins		
Staurosporine solution from *Streptomyces* sp	Sigma	Cat# S6942; MDL# MFCD04041131;
IMMPACT Dab Peroxidase (HRP) Substrate	Vector Laboratories	Cat# SK-4105; RRID:AB_2336520
REAL peroxidase blocking solution	Dako Agilent Pathology Solutions	Code: S2023
Lympholyte-H	Cedarlabs	Cat# CL5026
MitoTracker Red CMXRos	Thermo Fisher	Cat# M7512
pHrodo Red Dextran, 10,000 MW	Thermo Fisher	Cat# P10361
Fluorescein isothiocyanate–dextran 10,000 MW	Sigma	Cat# FD10S
Saponin	Sigma	Cat# S7900; CAS 8047-15-2
FM1-43 Dye	Thermo Fisher	Cat# T3163
Blebbistatin	Sigma	Cat# B0560, CAS 856925-71-8
Latrunculin A	Sigma	Cat# 428021; CAS 76343-93-6
Cytochalasin D	Sigma	Cat# C8273; CAS 22144-77-0
Wortmannin	Calbiochem	Cat# 681675; CAS 19545-26-7
H-1152	Tocris	Cat# 2414; CAS 871543-07-6

(*Continued on next page*)

Continued		
REAGENT or RESOURCE	SOURCE	IDENTIFIER
IL-2	Peprotech	Cat# 200-02
Y27632	Calbiochem	Cat# 688000; CAS 146986-50-7
Critical Commercial Assays		
EasySep Human CD4+CD127lowCD49d- Regulatory T Cell Enrichment Kit	StemCell Technologies, UK	Cat# 19232
EasySep Human $CD4^+CD25^-$	StemCell Technologies, UK	Custom-made
FITC Annexin V Apoptosis Detection Kit with 7-AAD	Biolegend	Cat# 640922
Experimental Models: Cell Lines		
Huh-7	Jane McKeating	RCB Cat# RCB1366; RRID:CVCL_0336
HepG2	ATCC	Cat# HB-8065; RRID:CVCL_0027
Raji	ATCC	Cat# CCL-86; RRID:CVCL_0511
Jurkat	ATCC	Clone E6.1; Cat# TIB-152; RRID:CVCL_0367
NKL	Marco Purbhoo	RRID:CVCL_0466
Software and Algorithms		
GraphPad PRISM v8.0	GraphPad software	https://www.graphpad.com/scientific-software/prism/
FlowJo software	FlowJo, LLC	RRID: SCR_008520
IMARIS v8.1.2	Bitplane	RRID: SCR_007370

LEAD CONTACT AND MATERIALS AVAILABILITY

Further information will be fulfilled by the Lead Contact, Zania Stamataki (z.stamataki@bham.ac.uk).

EXPERIMENTAL MODEL AND SUBJECT DETAILS

Human liver tissue

Human liver tissue was obtained from discarded explants from end-stage disease patients undergoing orthotopic liver transplantation or donor livers surplus to clinical requirements at the Queen Elizabeth Hospital, Birmingham. Tissue was used according to local research ethics committee approvals (06/Q702/61 South Birmingham, Birmingham, UK). Patient information is provided in Table S1.

Primary cell isolation

Lymphocytes were isolated from venous blood samples in EDTA from haemochromatosis patients at the Queen Elizabeth Hospital consented according to local research ethics committee approvals (04/Q2708/41 South Birmingham, Birmingham, UK). Peripheral blood mononuclear cells (PBMCs) were isolated from whole blood using Lympholyte cell separation media (Cedarlane labs, Canada). PMBCs were then washed with PBS and diluted to a concentration of $5x10^7$ in the recommended isolation media. $CD4^+$ T cells, $CD8^+$ T cells or $CD19^+$ B cells were isolated using EasySep magnetic negative selection kits (StemCell Technologies, UK), according to manufacturer's instructions. EasySep Human $CD4^+CD127^{low}CD49d^-$ kit was used for Treg isolation and a custom-made kit $CD4^+CD25^-$ for non-Treg cells was also prepared by StemCell Technologies. Primary human hepatocytes (PHH) were isolated as previously described (Pichard et al., 2006) and maintained in Williams E media (GIBCO, UK) for 24 hours. Cells were then switched to DMEM, supplemented with 32 mU/mL insulin, 2 μg/ml hydrocortisone, 20 mg/l Streptomycin and 0.4 mmol/l ornithine.

Cell lines

HepG2 cells (ATCC), Huh-7 and Huh-7-CD81-GFP cells (kind gift from Professor Jane McKeating, University of Oxford, UK) were maintained in Dulbecco's modification of Eagle's medium (DMEM; GIBCO) supplemented with 10% fetal bovine serum, 1% non-essential amino acids, 100 units/ml penicillin, 100 μg/ml streptomycin and 1% L-glutamine, at 37°C and 5% CO_2. Lymphoma cell lines Jurkat (ATCC, E6-1), Raji (ATCC CCL-86) and leukemia cell line NKL (Robertson et al., 1996; kind gift from Dr Marco Purbhoo, Imperial College London, UK) were maintained in RPMI 1640 (GIBCO) supplemented as for DMEM, at 37°C and 5% CO_2. NKL culture media was further supplemented with 500 IU IL-2 (Peprotech).

METHOD DETAILS

Paraffin-embedding of tissue

Liver wedge or biopsy samples from patients with chronic liver diseases, or non-cirrhotic donor livers were fixed before or after co-culture with peripheral blood-derived biotinylated $CD4^+$ T cells. All tissues were fixed in formalin for at least a week and embedded in paraffin wax. 3 μm-thick sections were then cut using a Leica RM2235 rotary microtome (Leica Biosystems, UK). Tissue sections were mounted onto charged glass slides. Sections were later de-waxed and stained by immunohistochemistry with a fluorescent or chromogenic readout.

Immunohistochemistry

All immunohistochemistry was performed using paraffin-embedded tissue. Tissue sections were deparaffinised with xylene, rehydrated using 97% industrial denatured alcohol (IDA) and then underwent antigen-retrieval by microwaving in antigen unmasking solution (Vector Laboratories, UK). Endogenous peroxidase activity and non-specific antibody binding was blocked using REAL peroxidase blocking solution (DAKO, UK) and 2X casein solution (Vector Labs), respectively. Sections were incubated with primary antibodies at room temperature for one hour. The following antibodies were used: pan-cytokeratin (Abcam, UK; ab27988), mouse anti-β-catenin (Biolegend, UK; 8446022), mouse anti-FOXP3 (Abcam; ab20034) and rabbit anti-Tbet (Santa-Cruz, USA; Sc-21003). Sections from tissues containing biotinylated $CD4^+$ T cells were also incubated with streptavidin-HRP (Vector Laboratories, SA-5004). Isotype-matched controls were used for all procedures. Primary antibodies were detected using anti-rabbit or anti-mouse HRP-conjugated secondary reagents (Vector Laboratories) and staining was developed using ImmPACT DAB (Vector Laboratories; SK-4105). Sections were counter-stained with Mayer's Haematoxylin (SigmaAldrich, UK), dehydrated and then mounted with DPX mountant (SigmaAldrich). Sections were then scanned using a Zeiss Axio Scan.Z1 slide scanner. For immunofluorescence, primary antibodies used were rabbit anti-CD3 (Abcam) and mouse anti-CD4 (Dako Agilent Pathology Solutions, UK), and mouse anti-β-catenin (Biolegend) and rabbit anti-Tbet (Santa-Cruz, USA; Sc-21003). Tissues were stained with conjugated secondary antibodies to AlexaFluor 488 or AlexaFluor 594 (Thermo Fisher Scientific). Autofluorescence was quenched using the TrueVIEW Autofluorescence Quenching Kit (Vector Labs). Tissues were then counterstained with 300 nM DAPI (4′,6-diamidino-2-phenylindole) and mounted. Tissues were imaged using the Zeiss LSM 780 or 880 microscope (Carl Zeiss Ltd) equipped with a x63 water immersion objective.

Co-culture assays

Hepatocytes and hepatoma cell lines were seeded on glass coverslips in 24-well plates or class-bottomed plates (MatTek) and allowed to adhere. Cells were then labeled with CellTracker Green (5-chloromethylfluorescein diacetate, CMFDA, Thermo Fisher Scientific) or Red (CMTPX, Thermo Fisher Scientific) and lymphocytes were labeled with a CellTracker Red (CMTPX) or Violet (2,3,6,7-tetrahydro-9- bromomethyl-1H,5H- quinolizino(9,1-gh)coumarin, BMQC, Thermo Fisher Scientific). Lymphocytes were layered on Lympholyte-H and centrifuged to remove dying cells.

Cell treatments and induction of cell death

Cells were treated with 1 μM staurosporine for 4 hours (primary cells) or overnight (lymphoma cells) to induce apoptosis. Necrosis was induced by heat-killing at 60°C, for 30 minutes. Cell death was confirmed by flow cytometry detection of labeling with 7-Aminoactinomycin D (7-AAD) and Annexin V (Biolegend). Primary T cell activation was performed with 0.5 μg/ml anti-CD3 (OKT3, Thermo Fisher, 16-0037-85) + 0.5 μg/ml anti-CD28 (Thermo Fisher, 16-0288-85) in the presence of 500 IU IL-2 (Peprotech, UK). The following inhibitors were used at the concentrations indicated in the figures: blebbistatin (SigmaAldrich), H1152 (Tocris), Y27632 (Calbiochem), wortmannin (Calbiochem), latrunculin A (SigmaAldrich), cytochalasin D (Thermo Fisher Scientific).

Cell-in-cell assays

Hepatocytes or hepatoma cell lines were cultured overnight and labeled with CellTracker dyes as stated. Lymphocytes were labeled with CellTracker dyes and rested in culture media. Live cells were separated from dead by gradient centrifugation. For efferocytosis assays, apoptotic or necrotic cells were induced as described. Labeled lymphocytes were co-cultured with hepatocytes at a ratio of 1:2 (hepatocyte: lymphocyte) for the times indicated at 37°C and 5% CO_2. Co-cultures were fixed and imaged using a Zeiss 780 or 880 confocal microscope and a x40 or x63 water objective.

Live cell imaging

To confirm internalised T cell viability, co-cultures were incubated with cell-permeable MitoTracker Red CMXRos (Thermo Fisher Scientific) which accumulates depending on active mitochondrial membrane potential. Acidification of phagosomes and enclytic vesicles was detected by pHrodo Red Dextran, 10,000 MW (Thermo Fisher Scientific) added to the culture media at 37°C. Fluorescein isothiocyanate–dextran 10,000 MW (SigmaAldrich) was also added to the media and cultured overnight, to measure endocytic trafficking to the enclytic vesicle. Complete T cell internalisation was confirmed with CellMask Deep Red or Orange plasma membrane stains or FM1-43 Dye (N-(3-Triethylammoniumpropyl)-4-(4-(Dibutylamino) Styryl) Pyridinium Dibromide) (Thermo Fisher Scientific).

Immunofluorescence

Cells were seeded on 13 mm glass coverslips. In most cases, cells were labeled with CellTracker dyes and/or Hoechst 33342 before fixation (Thermo Fisher Scientific). To detect surface or intracellular proteins, cells were stained before or after fixation/permeabilization, respectively. Cells were fixed with 3.7% formaldehyde, 1% paraformaldehyde or ice-cold methanol depending on the antigen detected. To permeabilize cells fixed in aldehyde-based fixatives, cells were washed in PBS and blocked for 30 min with 1% (w/v) FBS and 1% (w/v) BSA, in PBS, in the presence of 0.1% (w/v) saponin (SigmaAldrich). Primary antibodies were prepared in the same staining buffer and where relevant, saponin was present throughout the staining protocol. We used the following antibodies: anti-MRP2 (Abcam, ab110740), anti-LAMP-1 (BD Biosciences, 611042), anti-β-catenin (Biolegend), anti-E-cadherin (BD Bioscience, 610182), anti-ICAM-1 (R&D Systems,), anti-ICAM-2 (R&D Systems, AF244), anti-CD44 (Abcam, ab157107), anti-EpCAM (Biolegend, 324201), anti-β2 integrin (Novus Biologicals, AF1730), anti-β1 integrin (Novus Biologicals, MAB17781), Cells were incubated with primary antibody for one hour at room temperature (20-22°C), or 24 hours at 4°C. Cells were washed again in staining buffer and then incubated with secondary, fluorophore-conjugated antibodies in the same buffer. Cells were then washed twice in PBS and mounted on to glass microscope slides using Prolong gold antifade mounting reagent (Thermo Fisher Scientific). Coverslips were then imaged using confocal microscopy on the Zeiss LSM 780 or 880 and images were 3D volume-rendered using Bitplane IMARIS 8 cell biology software.

Scanning electron microscopy

Huh-7 cells were seed at a density of $5x10^4$ in 24-well plates on 12 mm glass coverslips and allowed 24 hours to adhere. Huh-7 cells were then co-cultured with live, apoptotic, or heat-killed primary $CD4^+$ T cells at $2.5x10^5$ T cells/well. Cells were co-cultured for 4 hours to ensure a selection of cells at different stages of internalisation at the point of fixation. Cells were washed with PBS to removed non-associating T cells and fixed in 2.5% (v/v) glutaraldehyde. Cells were then treated with osmium tetroxide and then imaged using a JSM-7000F (JEOL) SEM fitted with an Oxford Instruments Inca Energy Dispersive Spectroscopy (EDS) system, based at the Centre for Materials and Metallurgy at the University of Birmingham.

Time-lapse microscopy using the CQ1 High-Content Benchtop Microscope

Huh-7 cells were seeded at a density of 10^4 per well in black 96-well plates and allowed 24 hours to adhere. Huh-7 cells and $CD4^+$ T cells were labeled with CellTracker dyes. All media were replaced with pre-warmed CO_2-independent medium (GIBCO) containing 25 mM HEPES, 1% non-essential amino acids, 1% L-Glutamine, 1% Penicillin/Streptomycin and 10% Foetal Bovine Serum. For vesicle acidification assays, cells were co-cultured in the presence of 50 μg/mL pHrodo Red Dextran, 10,000 MW (Thermo Fisher Scientific). Equal numbers of live, apoptotic or heat-killed $CD4^+$ T cells were added and images were acquired every 15 minutes for 7 hours using a CQ1 High-Content Benchtop Microscope (Yokogawa). Cell-in-cell structures were quantified using ImageJ and IMARIS software.

Flow Cytometry

Huh-7 cells were labeled with CMFDA and incubated with blocking antibodies or isotype-matched controls, then co-cultured with CellTracker -labeled lymphocytes for three hours to allow enclysis. Co-cultures were dislodged from plastic using EDTA and analyzed using a CyAn ADP flow cytometer. Forward scatter (cell size), side scatter (cell internal complexity, granularity) and fluorescence were recorded and data analyzed using FlowJo software.

QUANTIFICATION AND STATISTICAL ANALYSIS

Graphs were made and statistical tests were performed using Graphpad Prism 8.0 software. The comparison of two groups of data was performed by Student's t tests, unpaired non-parametric data by Mann-Whitney test or for paired, non-parametric data by Wilcoxon test. Statistical significance was defined as p value < 0.05. Error bars on graphs represent standard deviations unless otherwise stated.

Graphical Abstract

Artwork was generated using free resources available through https://smart.servier.com/.

DATA AND CODE AVAILABILITY

This study did not generate any new datasets.

Cell Reports, Volume *29*

Supplemental Information

Hepatocytes Delete Regulatory T Cells by Enclysis,

a CD4$^+$ T Cell Engulfment Process

Scott P. Davies, Gary M. Reynolds, Alex L. Wilkinson, Xiaoyan Li, Rebecca Rose, Maanav Leekha, Yuxin S. Liu, Ratnam Gandhi, Emma Buckroyd, Joe Grove, Nicholas M. Barnes, Robin C. May, Stefan G. Hubscher, David H. Adams, Yuehua Huang, Omar Qureshi, and Zania Stamataki

Suppl. Figure 1.

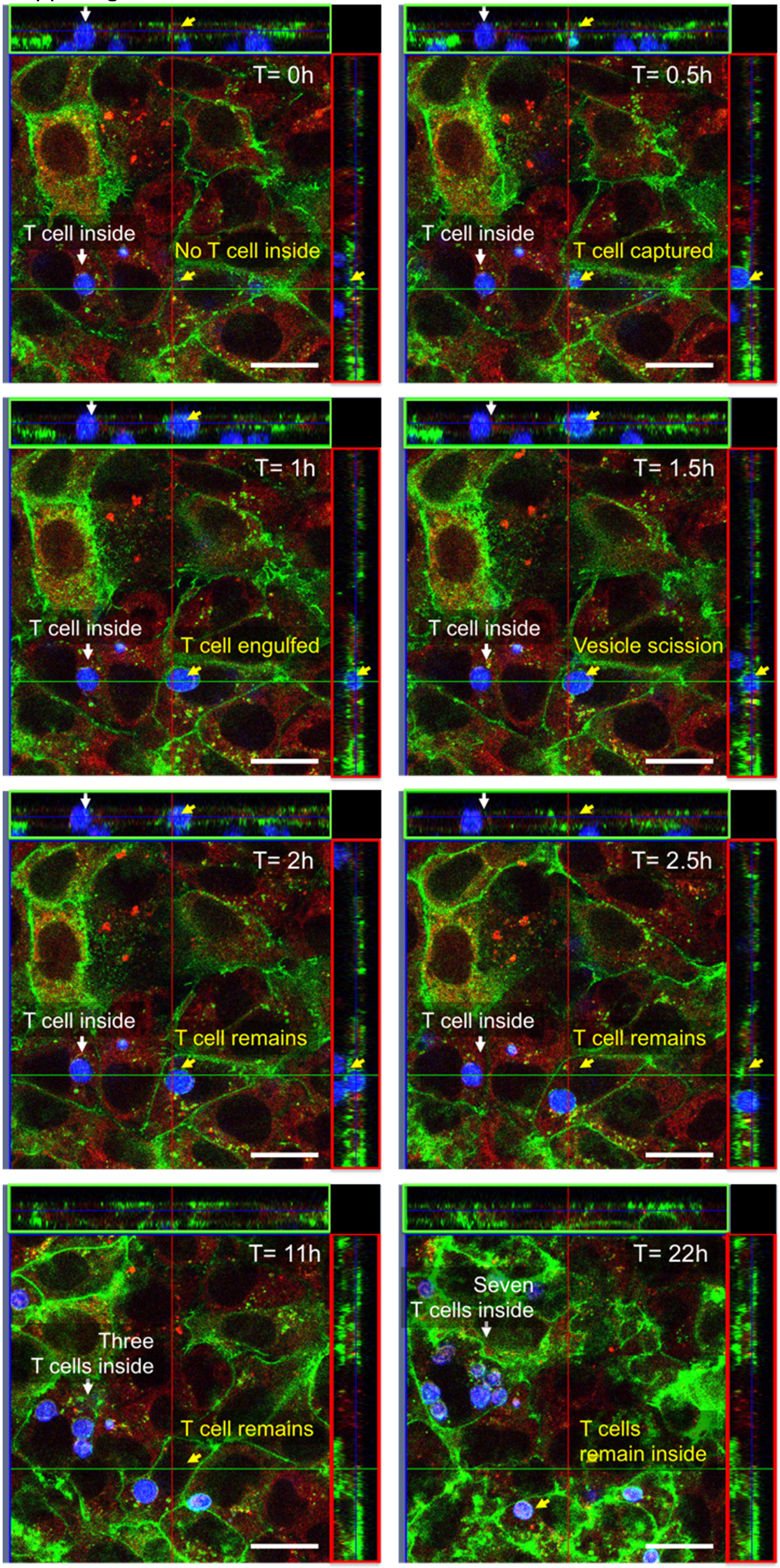

Suppl. Figure 1. Live $CD4^+$ T cells remained inside Huh-7 cells in vesicles that detached from the host cell membrane. Related to Figure 1. Time-lapse confocal imaging of Huh-7 cells (CMTPX, red cytoplasm) co-cultured with live $CD4^+$ T cells (BMQC, blue) over 22 hours. Huh-7 cell membranes were visualised with CD81-GFP (green). White arrow indicates a T cell that was fully internalised at the start of the imaging period (T=0). The consistent Z plane (blue line) was selected to confirm internalisation of the T cell, throughout the duration of the experiment. Orthogonal image coordinates (cross between green and red line) were set at the position where a new T cell appears, is captured and becomes engulfed within a vesicle that separated from the host cell membrane. Scale bars indicate 20 µm.

Suppl. Figure 2.

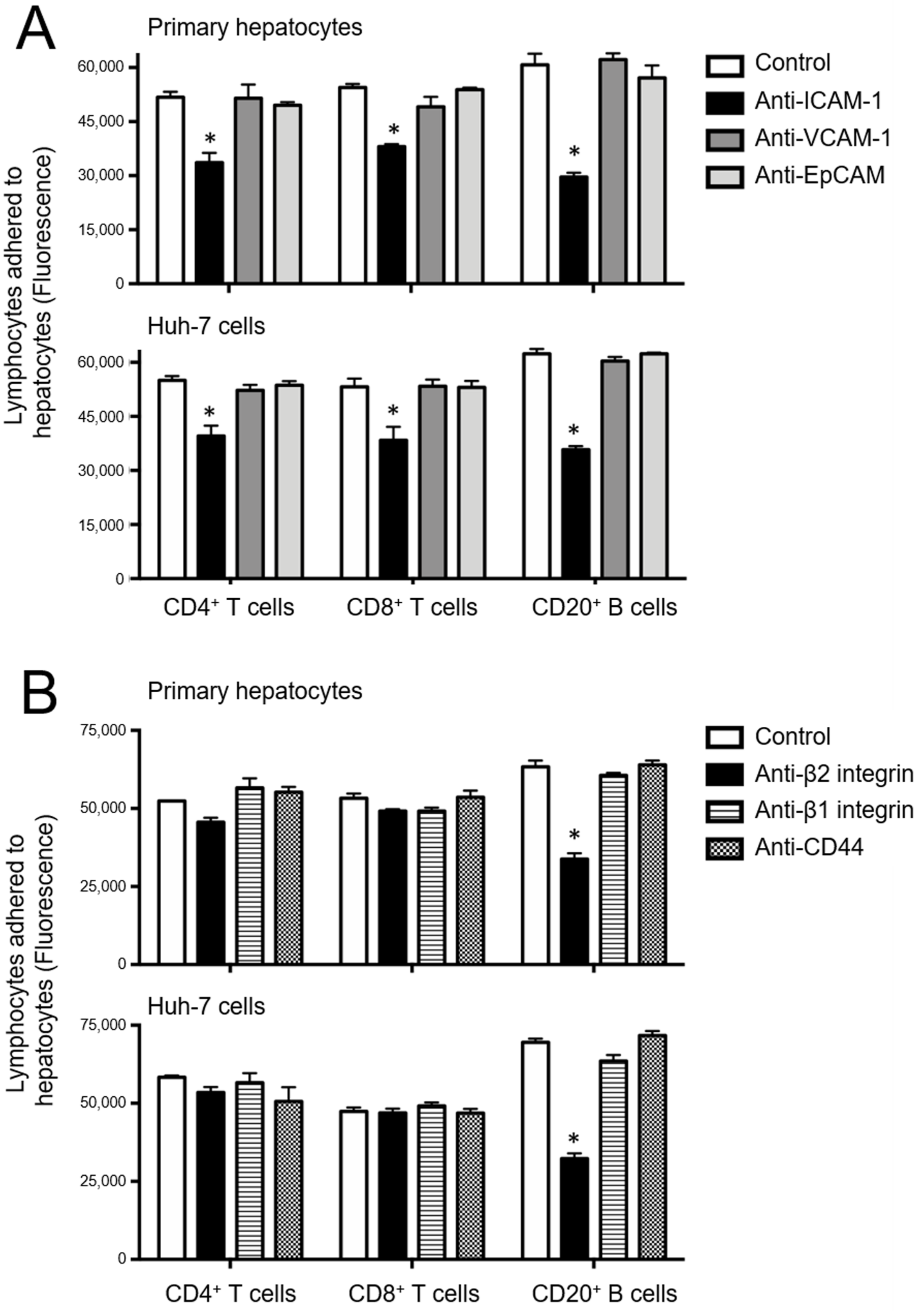

Suppl. Figure 2. Anti-ICAM-1 inhibits early lymphocyte adhesion to hepatocytes and Huh-7 cells. Related to Figure 2. Primary human hepatocytes or Huh-7 cells were seeded to confluence in 96-well plates and co-cultured with lymphocytes (CMFDA, green) for five minutes. **A.** Monolayers were treated with anti-ICAM-1, anti-VCAM-1 or anti-EpCAM or

isotype-matched control antibodies for 20 minutes before adding lymphocytes. **B.** Lymphocytes were treated with anti-β2 integrin, anti-β1 integrin, anti-CD44 or isotype-matched control antibodies before incubation with hepatocyte monolayers. Adhered lymphocytes persisting after washes were enumerated by total CMFDA fluorescence in replicate wells using a fluorescence plate reader. Data shown from four independent experiments with two primary hepatocyte donors and four lymphocyte donors. *$p < 0.05$, Student's non-parametric paired t test (Wilcoxon).

Suppl. Figure 3.

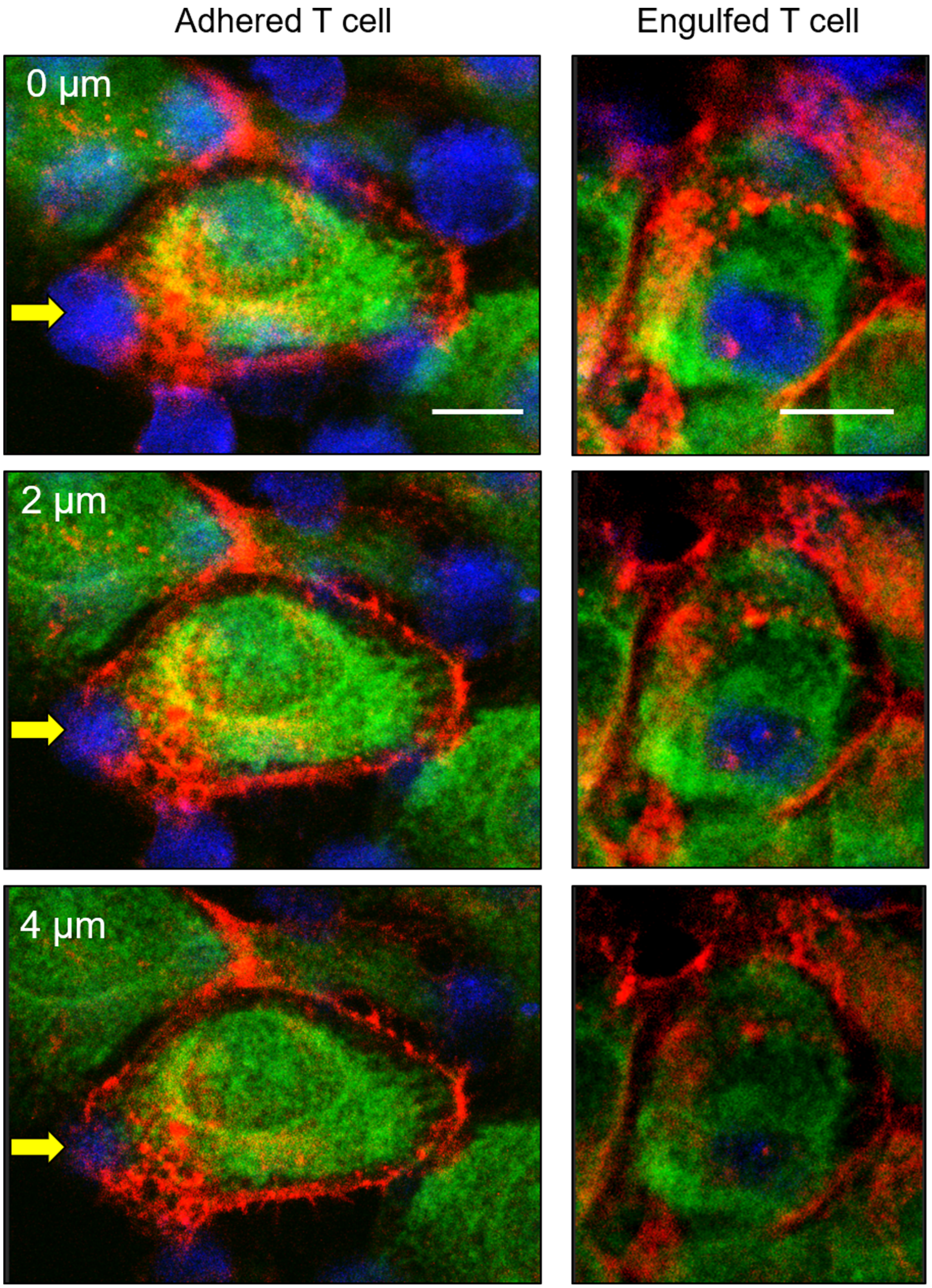

Suppl. Figure 3. Confocal z-stack images for ICAM-1 accumulation at the site of CD4$^+$ T cell adhesion and engulfment. Related to Figure 2. Huh-7 cells (CMFDA, green) were

co-cultured with Jurkat T cells (BMQC, blue) for three hours, fixed and stained for ICAM-1 (anti-mouse-Alexa 594, red), then imaged by confocal microscopy using a x63 objective. Images shown are 2 µm apart. The yellow arrow indicates membrane ICAM-1 accumulations around a T cell that has not been completely internalised (left panel, adhered T cell). The right panel shows minimal ICAM-1 expression around the engulfed T cell. Scale bar represents 10 µm.

Suppl. Figure 4.

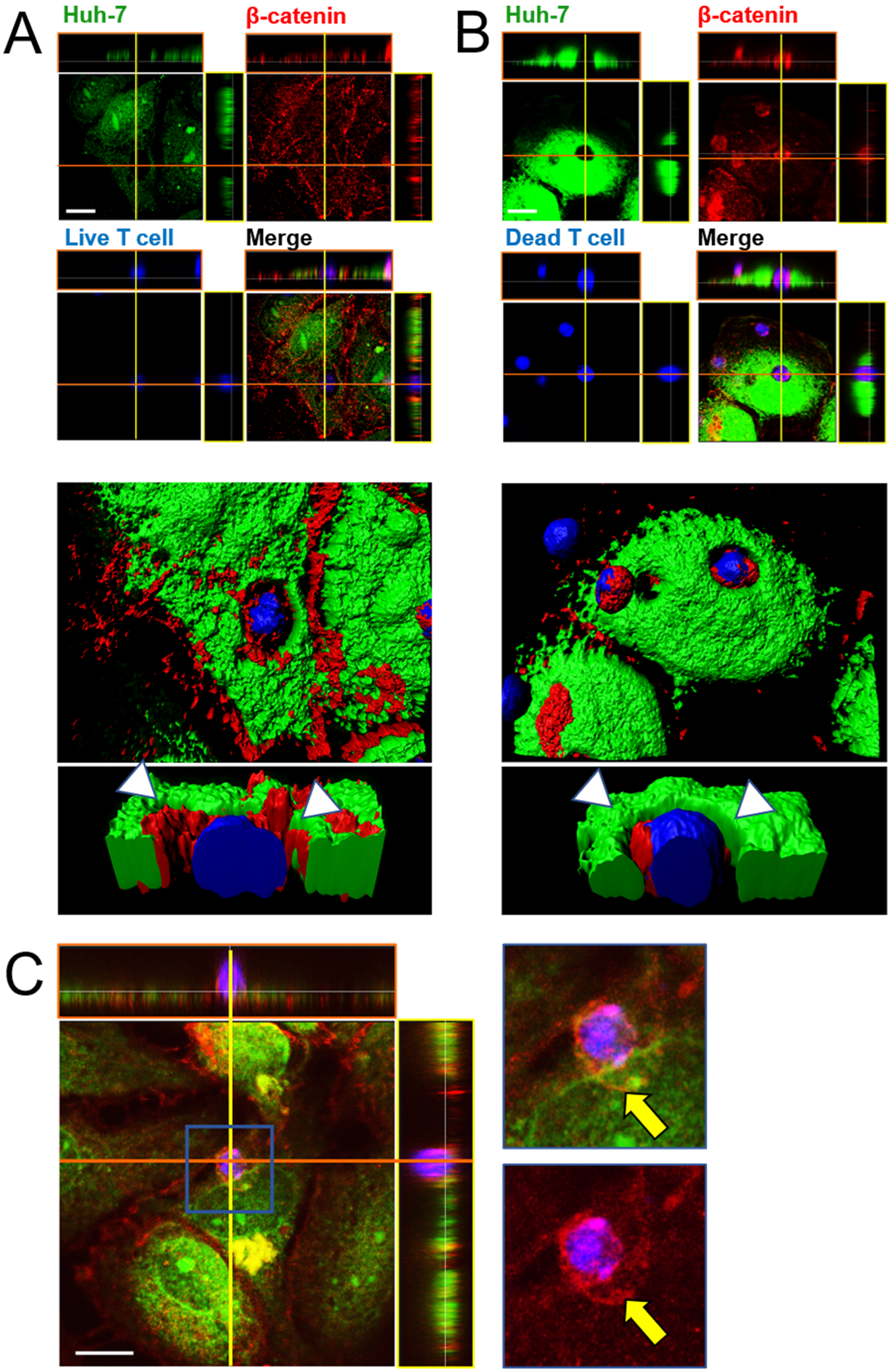

Suppl. Figure 4. Expression of β-catenin at the live T cell-containing vesicle and at the site of enclysis intermediates, but not in efferocytosis. **Related to Figure 4.** Huh-7 cells (CMFDA, green) were co-cultured with live or heat-killed Jurkat T cells (BMQC, blue) for three hours, fixed and stained for β-catenin (anti-mouse-Alexa 594, red), then imaged by confocal microscopy using a x63 objective. Non-rendered (Zeiss ZEN) and rendered (IMARIS) images are shown with white arrows showing the T cell-containing vesicle in **A.** enclysis and in **B.** efferocytosis. **C.** The presence β-catenin was also investigated in enclysis intermediates, where β-catenin accumulations were imaged on and around the adhered T cell (insets, yellow arrows). Scale bar represents 10 μm.

Suppl. Figure 5.

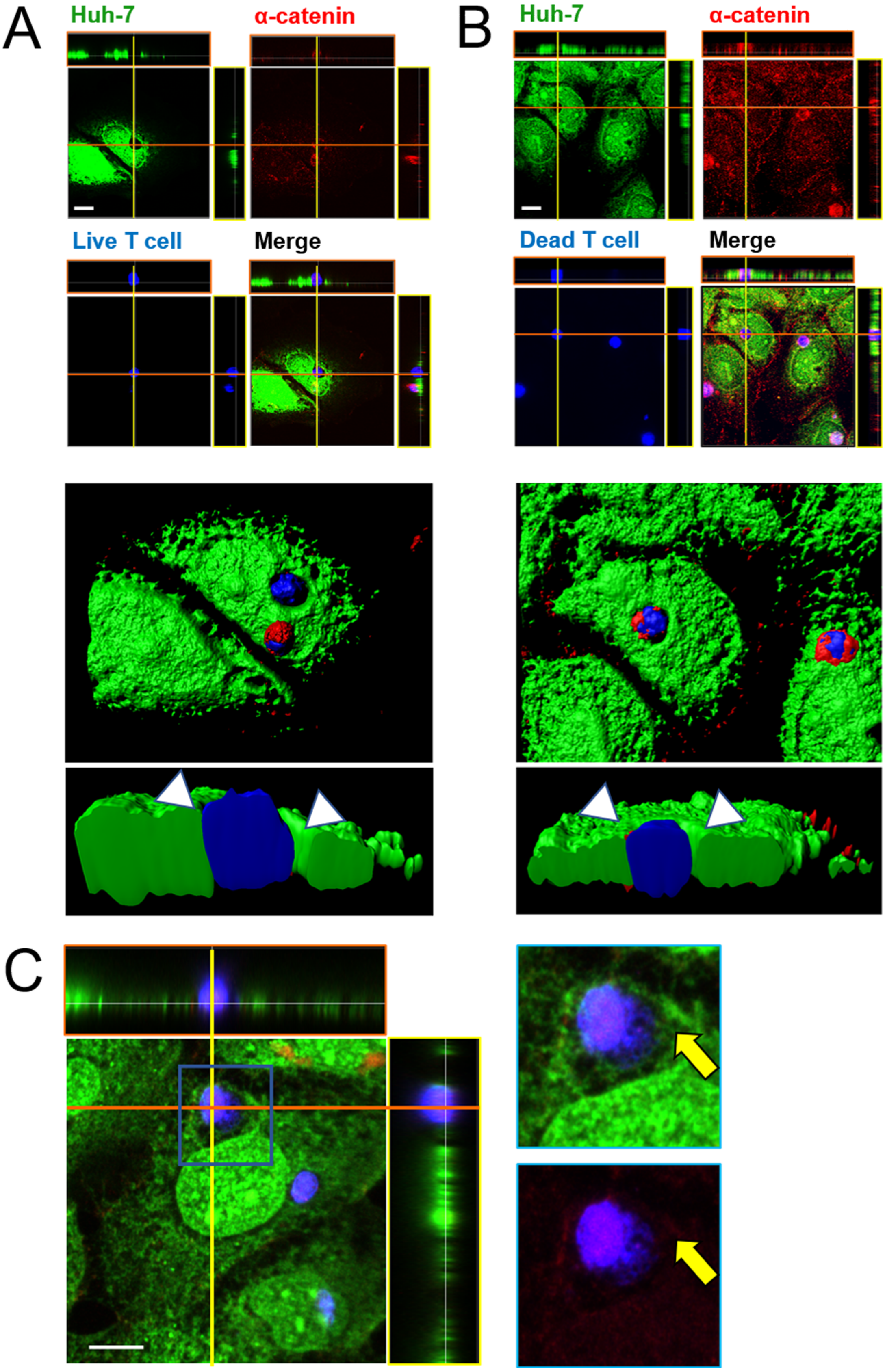

Suppl. Figure 5. α-catenin was not expressed at the site of enclysis intermediates and at the T cell-containing vesicle in enclysis or efferocytosis. **Related to Figure 4.** Huh-7 cells (CMFDA, green) were co-cultured with live or heat-killed Jurkat T cells (BMQC, blue) for three hours, fixed and stained for α-catenin (anti-mouse-Alexa 594, red), then imaged by confocal microscopy using a x63 objective. Non-rendered (Zeiss ZEN) and rendered (IMARIS) images are shown with white arrows showing the T cell-containing vesicle in **A.** enclysis and in **B.** efferocytosis. **C.** The presence α-catenin was also investigated in enclysis intermediates, where α-catenin did not accumulate around the adhered T cell (insets, yellow arrows). Scale bar represents 10 μm.

Suppl. Figure 6.

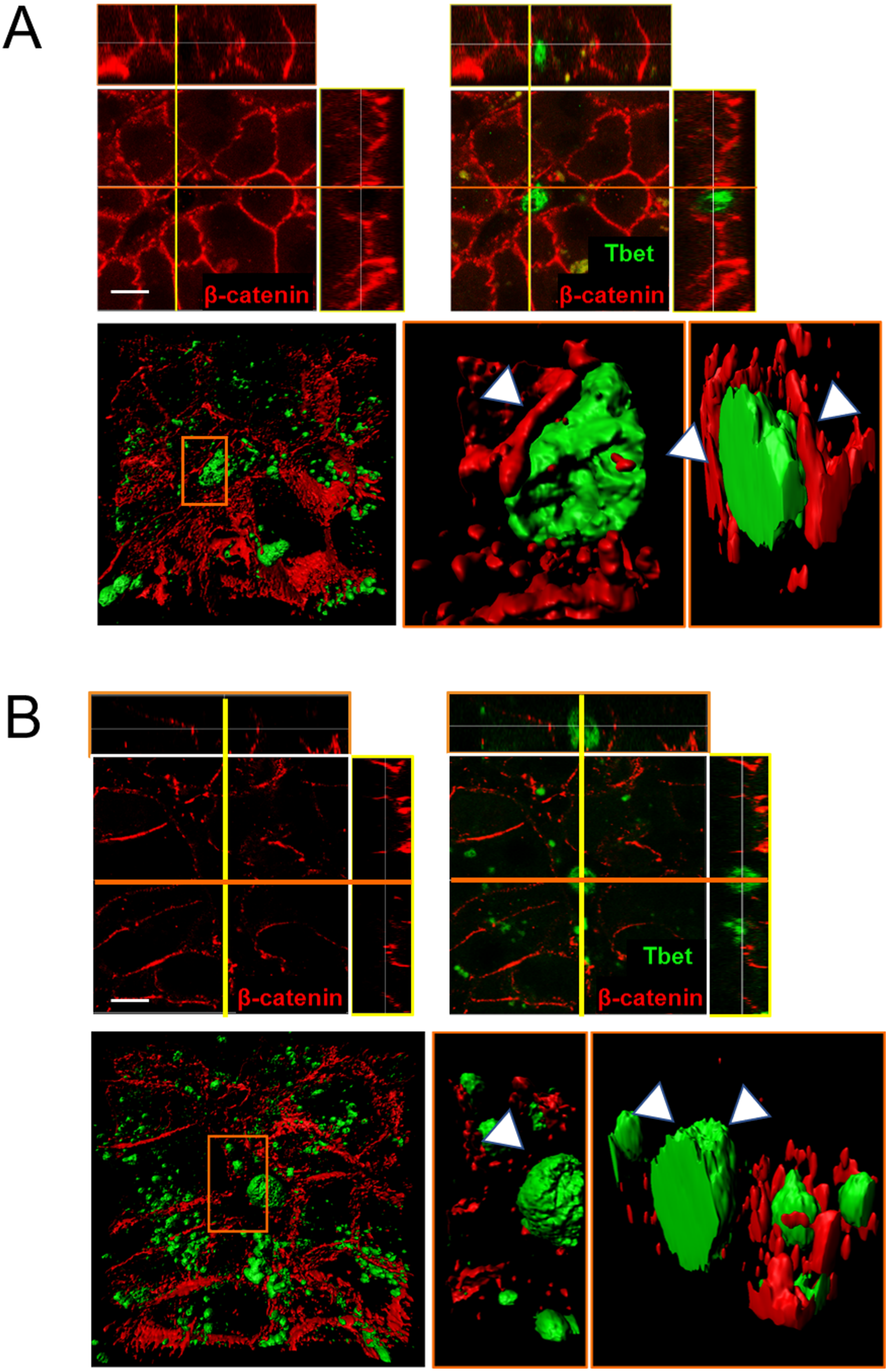

Suppl. Figure 6. Expression of β-catenin *in vivo* in end-stage liver disease. Related to Figure 4. Orthogonal confocal image of a 30 µm-thick section from formalin-fixed, paraffin-embedded tissue from a cirrhotic liver explant stained for β-catenin and $CD4^+$ T cell transcription factor Tbet. **A.** Hepatocyte containing a T cell where β-catenin molecules were observed at the enclytic vesicle (white arrows on 3-D rendered images by IMARIS). The hepatocyte membrane completely surrounds the enclosed T cell in the orthogonal view. **B.** Sinusoidal T cell for comparison, where there is no surrounding β-catenin association (white arrows on 3-D rendered images by IMARIS). Scale bar represents 10 µm.

Suppl. Figure 7.

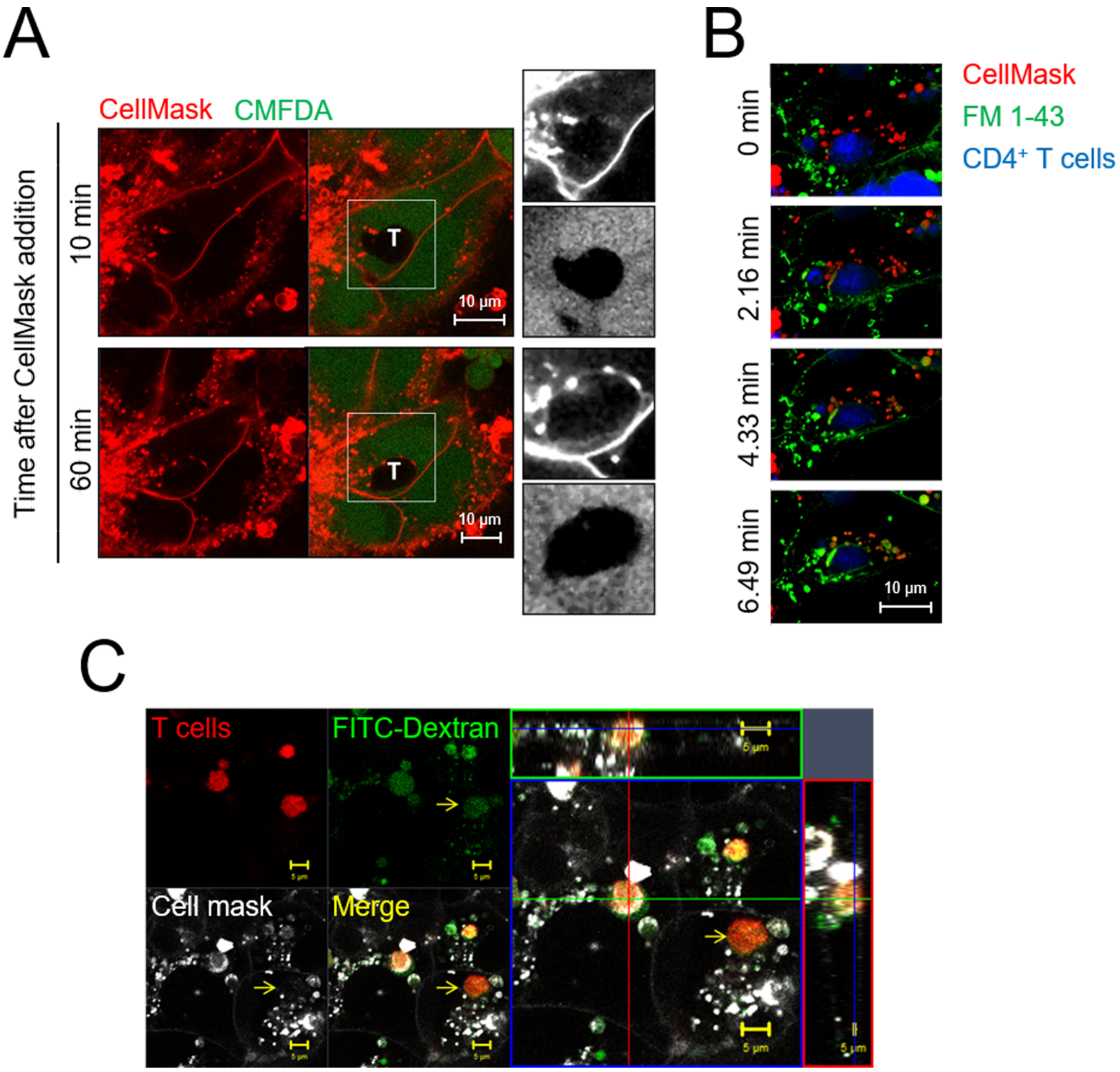

Suppl. Figure 7. Enclytic vesicles were connected to the endocytic pathway. Related to Figure 5. Live time-lapse confocal imaging detecting endocytic trafficking to the enclytic vesicle. **A.** Huh-7 cells (CMFDA, green) were co-cultured with $CD4^+$ T cells and enclytic vesicle detected (marked with a T), then CellMask™ Plasma Membrane Orange was applied to the culture media to reveal all accessible membranes. Imaged at 37°C, endosomes budding from the Huh-7 membrane (red) trafficked to the enclytic vesicle at 10 minutes following CellMask™ Plasma Membrane addition. Over time, endosomes fused with the enclytic vesicle membrane (60 minutes). **B.** Internalised T cell (BMQC, blue) was detected by CellMask™ Plasma Membrane Orange negative membrane (red) and FM 1-43 membrane dye (green). Within minutes (indicated), endocytic vesicles labelled by FM 1-43 trafficked to the T cell-containing vesicle and at six minutes the enclytic vesicle was labelled green. **C.** T cells inside Huh-7 cells were confirmed by lack of CellMask™ Plasma Membrane Deep Red staining. Fluorescent-labelled dextran (70kDa, FITC green) was added to the culture medium and the cells were imaged again following overnight incubation, when dextran had trafficked to the enclytic vesicle.

Suppl. Table 1. Patient information. Related to Figure 7. Age, sex and clinical information is given for patients used for immunohistochemistry measurements of enclysis. Patients were anonymised according to regulations approved by South Birmingham Research Ethics Committee. Donor livers: rejected for transplantation (non-cirrhotic). Autoimmune family disorders: autoimmune hepatitis (AIH), primary biliary cholangitis (PBC), primary sclerosing cholangitis (PSC). Metabolic injuries: alcohol liver disease (ALD), fatty liver disease (non-alcoholic steatohepatitis, NASH). Chronic viral infections: hepatitis B virus (HBV), hepatitis C virus (HCV). N/A: Not available.

	Donor	*AIH*	*PBC*	*PSC*	*ALD*	*NASH*	*HBV*	*HCV*
Cohort (n)	10	10	10	10	10	10	10	10
% female	N/A	50	0	30	40	50	10	10
Age Median	N/A	38.5	58	52	57	56	41.5	55.5
± *SD*		14.6	15.3	20.2	7.1	7.0	10.9	5.6
